# Supplementary material for: Animal Models for Studying Protein-Bound Uremic Toxin Removal—A Systematic Review
Source: Int J Mol Sci. 2023 Aug 25;24(17):13197. doi: 10.3390/ijms241713197 (PMC10487432; doi:10.3390/ijms241713197)
Supplement: Supplementary file 1 [file ijms-24-13197-s001.zip › Sup figure table_ proof.pdf]

Search string

((kidney diseases) OR (chronic kidney diseases)) AND ((uremic toxin) OR (protein-bound uremic toxin)) AND (animals))

Figures

Study or Subgroup	Year	Mean	SD	Total	Weight	Mean IV, Random, 95% CI
**Nephron loss**						
Aoki	2015	30.06	12.50	8	2.6%	30.06 [21.40; 38.72]
Aoyama	2002	39.78	0.00	14	2.8%	39.78 [39.78; 39.78]
Aoyama	2003	35.36	0.00	4	2.8%	35.36 [35.36; 35.36]
Bolati	2012	35.36	0.27	9	2.8%	35.36 [35.18; 35.54]
Deguchi	2005	67.01	8.21	15	2.8%	67.01 [62.86; 71.16]
Enomoto	2002	35.36	15.31	3	2.3%	35.36 [18.04; 52.68]
Huang	2018	32.00	4.50	6	2.8%	32.00 [28.40; 35.60]
Iwasaki	2013	44.20	0.00	6	2.8%	44.20 [44.20; 44.20]
Jerez-Morales	2021	17.72	5.79	5	2.7%	17.72 [12.64; 22.80]
Kikuchi	2017	62.77	5.30	5	2.8%	62.77 [58.12; 67.42]
Kobayashi	2002	88.40	0.00	9	2.8%	88.40 [88.40; 88.40]
Kuwahara	2014	25.64	2.65	6	2.8%	25.64 [23.52; 27.76]
Lekawanvijit	2012	40.25	13.79	12	2.7%	40.25 [32.45; 48.05]
Lin	2020	35.36	0.00	4	2.8%	35.36 [35.36; 35.36]
Miyazaki	2000	44.20	8.84	4	2.6%	44.20 [35.54; 52.86]
Owada	2007	47.74	8.67	24	2.8%	47.74 [44.27; 51.21]
Shi	2018	38.90	2.65	6	2.8%	38.90 [36.78; 41.02]
Shimoishi	2007	33.59	32.80	7	1.9%	33.59 [9.29; 57.89]
Tóthová	2015	57.84	26.20	15	2.5%	57.84 [44.58; 71.10]
Tumur	2008	40.66	6.49	6	2.7%	40.66 [35.47; 45.85]
Watanabe	2013	57.46	11.49	4	2.5%	57.46 [46.20; 68.72]
Zeng	2016	35.36	5.30	13	2.8%	35.36 [32.48; 38.24]
**Total (95% CI)**				**185**	**58.9%**	**43.08 [36.35; 49.80]**
Heterogeneity: $Tau^2 = 244.3523$; $Chi^2 = 16580628351.92$, df = 21 (P = 0); $I^2 = 100\%$						
**Tubular damage**						
Al Za'abi	2021	20.50	3.90	6	2.8%	20.50 [17.38; 23.62]
Al Za'abi	2021	17.45	2.55	6	2.8%	17.45 [15.41; 19.49]
Al Za'abi	2020	20.98	3.92	6	2.8%	20.98 [17.84; 24.12]
Ali	2018	13.20	0.73	6	2.8%	13.20 [12.62; 13.78]
Ali	2014	63.09	11.70	6	2.6%	63.09 [53.73; 72.45]
Ali	2019	17.10	2.57	6	2.8%	17.10 [15.04; 19.16]
Inami	2014	29.17	2.30	6	2.8%	29.17 [27.33; 31.01]
Iwata	2007	16.80	2.10	6	2.8%	16.80 [15.12; 18.48]
Kucey	2019	21.89	5.05	9	2.8%	21.89 [18.59; 25.19]
Kusumoto	2011	18.40	1.80	3	2.8%	18.40 [16.36; 20.44]
Matsushima	2017	29.17	12.25	3	2.4%	29.17 [15.31; 43.03]
Morisaki	2008	17.68	2.92	11	2.8%	17.68 [15.95; 19.41]
Sueyoshi	2019	24.50	0.01	12	2.8%	24.50 [24.49; 24.51]
Suleimani	2015	80.60	19.10	8	2.5%	80.60 [67.36; 93.84]
Won	2016	21.97	3.14	6	2.8%	21.97 [19.46; 24.48]
**Total (95% CI)**				**100**	**41.1%**	**26.85 [17.99; 35.71]**
Heterogeneity: $Tau^2 = 298.4583$; $Chi^2 = 1885.1$, df = 14 (P = 0); $I^2 = 99\%$						
**Total (95% CI)**				**285**	**100.0%**	**36.43 [30.48; 42.38]**
Heterogeneity: $Tau^2 = 328.3847$; $Chi^2 = 16666996577.99$, df = 36 (P = 0); $I^2 = 100\%$						
Test for subgroup differences: $Chi^2 = 8.17$, df = 1 (P < 0.01)						

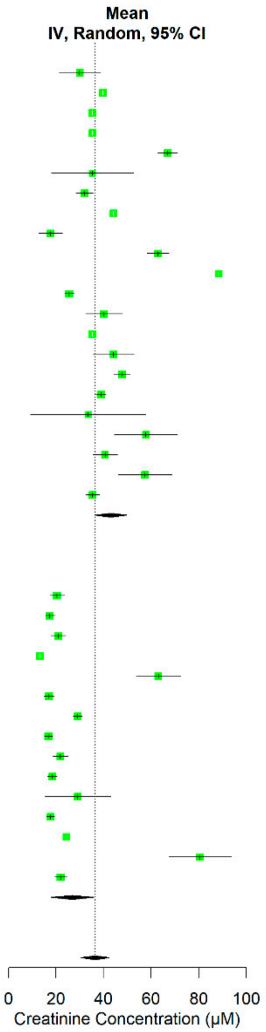

Study or Subgroup	Year	Mean	SD	Total	Weight	Mean IV, Random, 95% CI
Nephron loss						
Aoki	2015	93.70	25.00	8	3.0%	93.70 [76.38; 111.02]
Aoyama	2002	154.70	26.52	14	3.0%	154.70 [140.81; 168.59]
Aoyama	2003	159.12	59.31	5	2.2%	159.12 [107.13; 211.11]
Bolati	2012	124.64	57.50	8	2.5%	124.64 [84.80; 164.48]
Deguchi	2005	167.08	34.20	15	3.0%	167.08 [149.77; 184.39]
Enomoto	2002	159.12	23.40	7	3.0%	159.12 [141.79; 176.45]
Huang	2018	140.70	27.50	6	2.9%	140.70 [118.70; 162.70]
Iwasaki	2013	97.24	26.52	9	3.0%	97.24 [79.91; 114.57]
Jerez-Morales	2021	48.30	14.50	5	3.0%	48.30 [35.59; 61.01]
Kikuchi	2017	170.62	27.40	7	2.9%	170.62 [150.32; 190.92]
Kobayashi	2002	132.60	26.52	9	3.0%	132.60 [115.27; 149.93]
Kuwahara	2014	118.46	57.46	7	2.4%	118.46 [75.89; 161.03]
Lekawanvijit	2012	115.20	49.60	17	2.9%	115.20 [91.62; 138.78]
Lin	2020	114.92	17.68	5	3.0%	114.92 [99.42; 130.42]
Miyazaki	2000	132.60	17.38	5	3.0%	132.60 [117.37; 147.83]
Owada	2007	137.02	120.20	10	1.7%	137.02 [62.52; 211.52]
Shi	2018	176.80	12.40	6	3.0%	176.80 [166.88; 186.72]
Shimoishi	2007	173.27	35.08	7	2.8%	173.27 [147.28; 199.26]
Tóthová	2015	197.70	282.60	15	0.8%	197.70 [54.69; 340.71]
Tumur	2008	161.78	82.30	6	1.9%	161.78 [95.93; 227.63]
Watanabe	2013	113.15	21.22	11	3.0%	113.15 [100.61; 125.69]
Zeng	2016	85.75	10.61	9	3.1%	85.75 [78.82; 92.68]
Total (95% CI)				191	58.8%	131.42 [116.09; 146.76]
Heterogeneity: $Tau^2 = 1109.4955$; $Chi^2 = 484.27$, df = 21 ($P < 0.01$); $I^2 = 96\%$						
Tubular damage						
Al Za'abi	2021	58.20	12.50	6	3.0%	58.20 [48.20; 68.20]
Al Za'abi	2021	57.82	9.41	6	3.1%	57.82 [50.29; 65.35]
Al Za'abi	2020	141.20	47.08	6	2.5%	141.20 [103.53; 178.87]
Ali	2018	67.10	9.10	6	3.1%	67.10 [59.82; 74.38]
Ali	2014	205.57	30.50	6	2.8%	205.57 [181.17; 229.97]
Ali	2019	83.30	14.50	6	3.0%	83.30 [71.70; 94.90]
Inami	2014	138.79	49.50	6	2.5%	138.79 [99.18; 178.40]
Iwata	2007	171.10	23.80	6	2.9%	171.10 [152.06; 190.14]
Kucey	2019	207.90	71.30	12	2.5%	207.90 [167.56; 248.24]
Kusumoto	2011	99.90	17.10	3	2.9%	99.90 [80.55; 119.25]
Matsushima	2017	197.10	62.70	3	1.8%	197.10 [126.15; 268.05]
Morisaki	2008	130.83	43.98	11	2.8%	130.83 [104.84; 156.82]
Sueyoshi	2019	190.00	75.90	11	2.4%	190.00 [145.15; 234.85]
Suleimani	2015	136.80	36.80	6	2.7%	136.80 [107.35; 166.25]
Won	2016	76.40	8.37	6	3.1%	76.40 [69.70; 83.10]
Total (95% CI)				100	41.2%	127.50 [99.79; 155.21]
Heterogeneity: $Tau^2 = 2768.1520$; $Chi^2 = 378.5$, df = 14 ($P < 0.01$); $I^2 = 96\%$						
**Total (95% CI)**				**291**	**100.0%**	**129.50 [115.23; 143.77]**
Heterogeneity: $Tau^2 = 1712.6658$; $Chi^2 = 1133.36$, df = 36 ($P < 0.01$); $I^2 = 97\%$						
Test for subgroup differences: $Chi^2 = 0.06$, df = 1 ($P = 0.81$)						

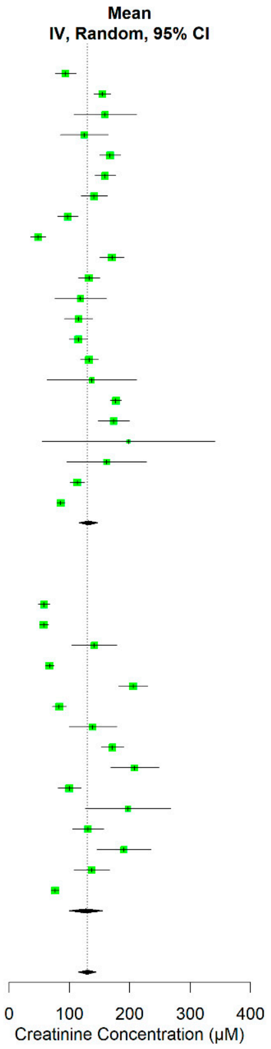

Figure S1. One-sided Forest plots of creatinine concentrations (μM) in healthy (top) and diseased (bottom) rats stratified by damage type.

Study or Subgroup	Year	Mean	SD	Total	Weight	Mean IV, Random, 95% CI
**Nephron loss**						
Aoki	2015	6.30	0.81	8	3.3%	6.30 [5.74; 6.86]
Aoyama	2002	10.34	0.36	14	3.3%	10.34 [10.15; 10.53]
Aoyama	2003	8.93	2.14	4	3.1%	8.93 [6.83; 11.03]
Deguchi	2005	5.50	0.70	15	3.3%	5.50 [5.15; 5.85]
Enomoto	2002	8.21	0.62	3	3.3%	8.21 [7.51; 8.91]
Iwasaki	2013	6.50	0.36	6	3.3%	6.50 [6.21; 6.79]
Kobayashi	2002	24.30	3.30	9	3.1%	24.30 [22.14; 26.46]
Kuwahara	2014	7.46	0.20	6	3.3%	7.46 [7.30; 7.62]
Lin	2020	4.18	0.40	4	3.3%	4.18 [3.79; 4.57]
Ling Lau	2019	7.18	0.39	5	3.3%	7.18 [6.84; 7.52]
Miyazaki	2000	7.14	0.71	4	3.3%	7.14 [6.44; 7.84]
Shi	2018	9.33	0.12	6	3.3%	9.33 [9.23; 9.43]
Shimoishi	2007	5.71	5.68	7	2.7%	5.71 [1.50; 9.92]
Tóthová	2015	9.66	3.37	15	3.2%	9.66 [7.95; 11.37]
Tumur	2008	6.07	0.87	6	3.3%	6.07 [5.37; 6.77]
Watanabe	2013	6.70	0.70	4	3.3%	6.70 [6.01; 7.39]
Zeng	2016	6.42	4.82	13	3.0%	6.42 [3.80; 9.04]
**Total (95% CI)**				**129**	**55.1%**	**8.21 [6.15; 10.26]**
Heterogeneity: Tau^2 = 18.1479; Chi^2 = 2165.66, df = 16 (P = 0); I^2 = 99%						
**Tubular damage**						
Al Za'abi	2021	3.00	1.50	6	3.3%	3.00 [1.80; 4.20]
Al Za'abi	2021	3.73	0.61	6	3.3%	3.73 [3.24; 4.22]
Al Za'abi	2020	6.13	3.77	6	2.9%	6.13 [3.11; 9.15]
Ali	2018	4.40	0.24	6	3.3%	4.40 [4.21; 4.59]
Ali	2014	6.36	1.96	6	3.2%	6.36 [4.79; 7.93]
Ali	2019	3.70	0.49	6	3.3%	3.70 [3.31; 4.09]
Inami	2014	8.90	1.39	6	3.3%	8.90 [7.79; 10.01]
Iwata	2007	7.18	2.62	6	3.1%	7.18 [5.08; 9.28]
Kucey	2019	6.44	2.14	9	3.2%	6.44 [5.04; 7.84]
Kusumoto	2011	9.32	2.13	3	3.1%	9.32 [6.91; 11.73]
Morisaki	2008	7.80	1.18	11	3.3%	7.80 [7.10; 8.50]
Sueyoshi	2019	7.05	0.01	12	3.3%	7.05 [7.04; 7.06]
Suleimani	2015	22.80	5.10	8	2.8%	22.80 [19.27; 26.33]
Won	2016	7.40	0.94	6	3.3%	7.40 [6.65; 8.15]
**Total (95% CI)**				**97**	**44.9%**	**7.29 [4.98; 9.60]**
Heterogeneity: Tau^2 = 18.7727; Chi^2 = 1329.51, df = 13 (P < 0.01); I^2 = 99%						
**Total (95% CI)**				**226**	**100.0%**	**7.79 [6.27; 9.31]**
Heterogeneity: Tau^2 = 18.0053; Chi^2 = 5284.03, df = 30 (P = 0); I^2 = 99%						
Test for subgroup differences: Chi^2 = 0.34, df = 1 (P = 0.56)						

Mean IV, Random, 95% CI

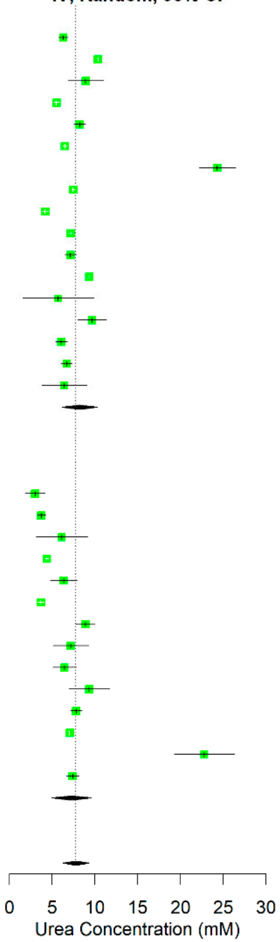

Study or Subgroup	Year	Mean	SD	Total	Weight	Mean IV, Random, 95% CI
Nephron loss						
Aoki	2015	25.30	7.70	8	3.3%	25.30 [19.96; 30.64]
Aoyama	2002	27.13	0.71	14	3.3%	27.13 [26.76; 27.50]
Aoyama	2003	33.57	9.58	5	3.2%	33.57 [25.17; 41.97]
Deguchi	2005	15.40	1.80	15	3.3%	15.40 [14.49; 16.31]
Enomoto	2002	36.07	4.72	7	3.3%	36.07 [32.57; 39.57]
Iwasaki	2013	15.78	3.96	9	3.3%	15.78 [13.19; 18.37]
Kobayashi	2002	32.10	4.20	9	3.3%	32.10 [29.36; 34.84]
Kuwahara	2014	25.80	6.20	7	3.3%	25.80 [21.21; 30.39]
Lin	2020	22.40	4.86	5	3.3%	22.40 [18.14; 26.66]
Ling Lau	2019	57.50	18.50	4	3.0%	57.50 [39.37; 75.63]
Miyazaki	2000	24.64	2.85	5	3.3%	24.64 [22.14; 27.14]
Shi	2018	54.60	22.40	6	3.0%	54.60 [36.68; 72.52]
Shimoishi	2007	36.10	13.20	7	3.2%	36.10 [26.32; 45.88]
Tóthová	2015	28.15	47.50	15	2.7%	28.15 [4.11; 52.19]
Tumur	2008	30.85	15.10	6	3.1%	30.85 [18.77; 42.93]
Watanabe	2013	16.70	4.00	11	3.3%	16.70 [14.34; 19.06]
Zeng	2016	40.71	9.20	9	3.3%	40.71 [34.70; 46.72]
**Total (95% CI)**				**142**	**54.6%**	**29.06 [24.20; 33.91]**
Heterogeneity: $Tau^2 = 87.7265$; $Chi^2 = 767.46$, df = 16 ($P < 0.01$); $I^2 = 98\%$						
Tubular damage						
Al Za'abi	2021	18.00	3.85	6	3.3%	18.00 [14.92; 21.08]
Al Za'abi	2021	16.41	2.42	6	3.3%	16.41 [14.47; 18.35]
Al Za'abi	2020	42.94	15.40	6	3.1%	42.94 [30.62; 55.26]
Ali	2018	14.90	1.47	6	3.3%	14.90 [13.72; 16.08]
Ali	2014	150.71	7.08	6	3.3%	150.71 [145.04; 156.38]
Ali	2019	22.00	3.40	6	3.3%	22.00 [19.28; 24.72]
Inami	2014	40.50	7.80	6	3.3%	40.50 [34.26; 46.74]
Iwata	2007	59.90	10.50	6	3.2%	59.90 [51.50; 68.30]
Kucey	2019	68.20	12.00	12	3.3%	68.20 [61.41; 74.99]
Kusumoto	2011	42.62	4.80	3	3.3%	42.62 [37.19; 48.05]
Morisaki	2008	48.57	11.96	11	3.3%	48.57 [41.50; 55.64]
Sueyoshi	2019	45.99	16.86	11	3.2%	45.99 [36.03; 55.95]
Suleimani	2015	77.50	25.50	6	2.9%	77.50 [57.10; 97.90]
Won	2016	28.50	1.25	6	3.3%	28.50 [27.50; 29.50]
**Total (95% CI)**				**97**	**45.4%**	**48.13 [29.59; 66.66]**
Heterogeneity: $Tau^2 = 1235.1742$; $Chi^2 = 2701.23$, df = 13 ($P = 0$); $I^2 = 100\%$						
**Total (95% CI)**				**239**	**100.0%**	**38.39 [29.07; 47.72]**

Heterogeneity: $Tau^2 = 681.4240$; $Chi^2 = 3469.60$, df = 30 ($P = 0$); $I^2 = 99\%$

Test for subgroup differences: $Chi^2 = 3.80$, df = 1 ($P = 0.05$)

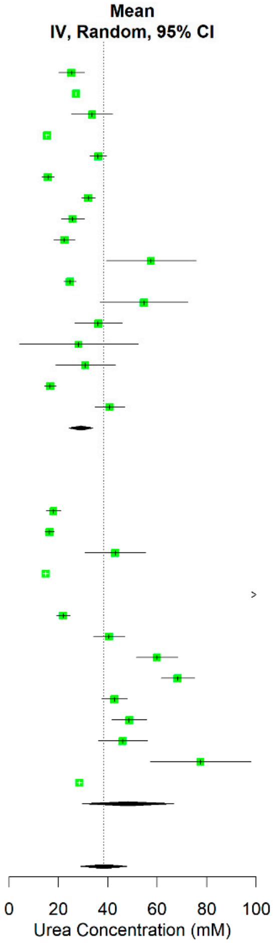

Figure S2. One-sided forest plots of urea concentrations (mM) in healthy (top) and diseased (bottom) rats stratified by damage type.

Study or Subgroup	Year	Mean	SD	Total	Weight	Mean IV, Random, 95% CI
**Nephron loss**						
Aoki	2015	3.10	1.13	8	4.4%	3.10 [2.32; 3.88]
Aoyama	2002	1.80	0.12	14	5.2%	1.80 [1.74; 1.86]
Aoyama	2003	3.08	0.74	4	4.5%	3.08 [2.35; 3.81]
Bolati	2012	3.68	0.39	9	5.1%	3.68 [3.43; 3.93]
Deguchi	2005	2.83	0.35	15	5.2%	2.83 [2.65; 3.01]
Enomoto	2002	2.59	0.12	3	5.2%	2.59 [2.45; 2.73]
Iwasaki	2013	2.80	0.20	6	5.2%	2.80 [2.64; 2.96]
Kikuchi	2017	2.99	0.32	5	5.1%	2.99 [2.71; 3.27]
Kuwahara	2014	4.48	0.42	6	5.1%	4.48 [4.14; 4.82]
Miyazaki	2000	2.15	0.23	4	5.2%	2.15 [1.92; 2.38]
Owada	2007	1.99	0.78	24	5.1%	1.99 [1.68; 2.30]
Shimoishi	2007	2.40	0.48	7	5.0%	2.40 [2.04; 2.76]
Tumur	2008	3.40	0.18	6	5.2%	3.40 [3.26; 3.55]
Watanabe	2013	2.46	0.96	4	4.2%	2.46 [1.52; 3.40]
**Total (95% CI)**				**115**	**69.7%**	**2.84 [2.45; 3.22]**
Heterogeneity: $Tau^2 = 0.4966$; $Chi^2 = 862.61$, df = 13 ($P < 0.01$); $I^2 = 98\%$						
**Tubular damage**						
Al Za'abi	2021	1.77	0.54	6	5.0%	1.77 [1.34; 2.20]
Al Za'abi	2021	2.10	0.29	6	5.1%	2.10 [1.87; 2.33]
Al Za'abi	2020	2.47	0.69	6	4.8%	2.47 [1.92; 3.02]
Ali	2014	1.06	0.49	6	5.0%	1.06 [0.67; 1.45]
Ali	2019	2.20	0.24	6	5.2%	2.20 [2.01; 2.39]
Suleimani	2015	0.06	0.02	8	5.2%	0.06 [0.05; 0.08]
**Total (95% CI)**				**38**	**30.3%**	**1.60 [0.87; 2.33]**
Heterogeneity: $Tau^2 = 0.8039$; $Chi^2 = 920.21$, df = 5 ($P < 0.01$); $I^2 = 99\%$						
**Total (95% CI)**				**153**	**100.0%**	**2.46 [2.04; 2.89]**

Heterogeneity: $Tau^2 = 0.8890$; $Chi^2 = 11024.90$, df = 19 ($P = 0$); $I^2 = 100\%$

Test for subgroup differences: $Chi^2 = 8.65$, df = 1 ($P < 0.01$)

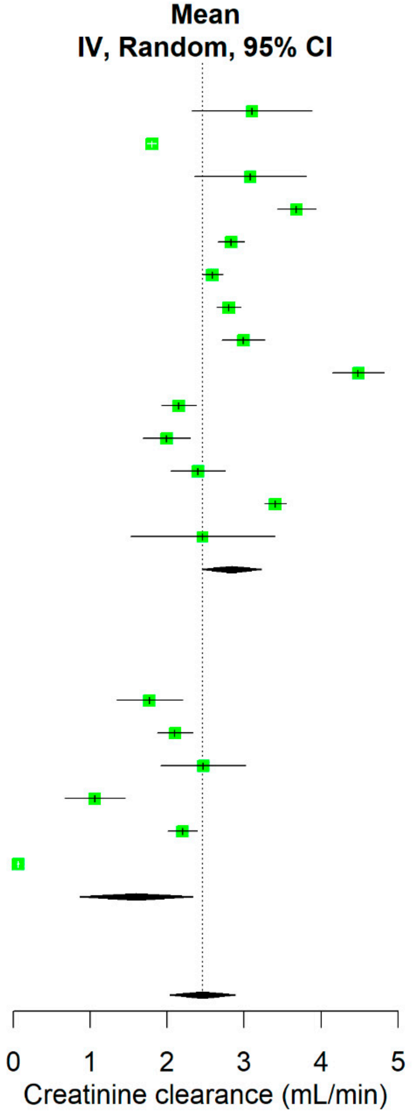

Study or Subgroup	Year	Mean	SD	Total	Weight	Mean IV, Random, 95% CI
**Nephron loss**						
Aoki	2015	0.88	0.25	8	5.1%	0.88 [0.71; 1.05]
Aoyama	2002	0.63	0.06	14	5.3%	0.63 [0.60; 0.66]
Aoyama	2003	0.72	0.27	5	4.9%	0.72 [0.49; 0.95]
Bolati	2012	1.14	0.45	8	4.6%	1.14 [0.83; 1.45]
Deguchi	2005	1.58	0.23	15	5.2%	1.58 [1.46; 1.70]
Enomoto	2002	0.76	0.18	7	5.1%	0.76 [0.62; 0.90]
Iwasaki	2013	1.10	0.30	9	5.0%	1.10 [0.90; 1.30]
Kikuchi	2017	1.11	0.16	7	5.2%	1.11 [0.99; 1.23]
Kuwahara	2014	1.28	0.53	7	4.2%	1.28 [0.89; 1.67]
Miyazaki	2000	0.65	0.12	5	5.2%	0.65 [0.54; 0.76]
Owada	2007	0.45	0.19	10	5.2%	0.45 [0.33; 0.57]
Shimoishi	2007	0.72	0.56	7	4.1%	0.72 [0.31; 1.13]
Tumur	2008	0.83	0.49	6	4.2%	0.83 [0.44; 1.22]
Watanabe	2013	0.66	0.12	11	5.3%	0.66 [0.59; 0.73]
**Total (95% CI)**				**119**	**68.5%**	**0.89 [0.72; 1.05]**
Heterogeneity: $Tau^2 = 0.0906$; $Chi^2 = 338.35$, df = 13 ($P < 0.01$); $I^2 = 96\%$						
**Tubular damage**						
Al Za'abi	2021	0.44	0.20	6	5.1%	0.44 [0.28; 0.60]
Al Za'abi	2021	0.35	0.05	6	5.3%	0.35 [0.31; 0.39]
Al Za'abi	2020	0.18	0.07	6	5.3%	0.18 [0.12; 0.24]
Ali	2014	0.29	0.10	6	5.2%	0.29 [0.21; 0.37]
Ali	2019	0.20	0.00	6	5.3%	0.20 [0.20; 0.20]
Suleimani	2015	0.03	0.01	6	5.3%	0.03 [0.02; 0.04]
**Total (95% CI)**				**36**	**31.5%**	**0.24 [0.13; 0.35]**
Heterogeneity: $Tau^2 = 0.0182$; $Chi^2 = 2236.94$, df = 5 ($P = 0$); $I^2 = 100\%$						
**Total (95% CI)**				**155**	**100.0%**	**0.69 [0.51; 0.87]**
Heterogeneity: $Tau^2 = 0.1575$; $Chi^2 = 4296.48$, df = 19 ($P = 0$); $I^2 = 100\%$						
Test for subgroup differences: $Chi^2 = 39.54$, df = 1 ($P < 0.01$)						

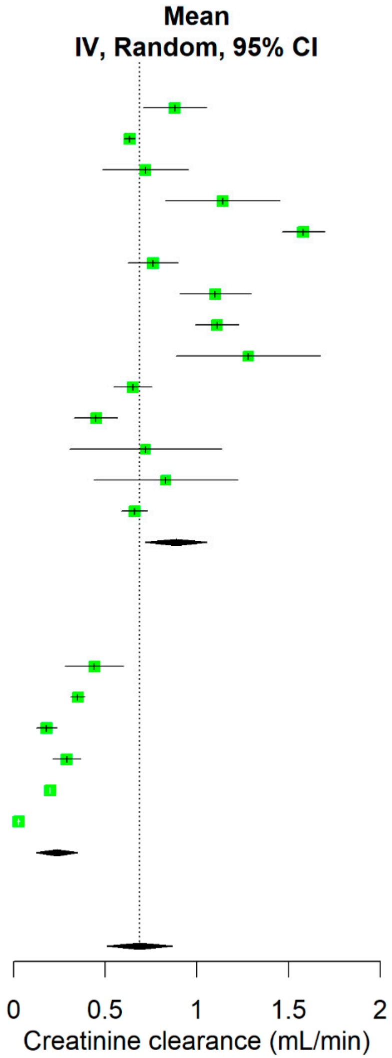

Figure S3. One-sided forest plots of creatinine clearance (mL/min) in healthy (top) and diseased (bottom) rats stratified by damage type.

Study or Subgroup	Year	Mean	SD	Total	Weight	Mean IV, Random, 95% CI
**Nephron loss**						
Dou	2017	31.90	3.60	6	7.8%	31.90 [29.02; 34.78]
Hung	2016	56.60	31.90	9	5.8%	56.60 [35.76; 77.44]
Makhloufi	2020	32.40	3.10	8	7.9%	32.40 [30.25; 34.55]
Ni	2014	1.31	0.11	6	7.9%	1.31 [1.22; 1.40]
Tungasanga	2022	13.70	0.01	5	7.9%	13.70 [13.69; 13.71]
Yamada	2019	8.84	0.45	5	7.9%	8.84 [8.45; 9.23]
**Total (95% CI)**				**39**	**45.1%**	**22.64 [7.94; 37.34]**
Heterogeneity: $Tau^2 = 322.4681$; $Chi^2 = 76395.07$, df = 5 (P = 0); $I^2 = 100\%$						
**Tubular damage**						
Makhloufi	2020	27.50	3.68	8	7.8%	27.50 [24.95; 30.05]
Mishima	2017	5.06	0.75	4	7.9%	5.06 [4.33; 5.79]
Mishima	2018	9.00	0.60	6	7.9%	9.00 [8.52; 9.48]
Nanto-Hara	2020	11.70	2.40	9	7.9%	11.70 [10.13; 13.27]
Shiba	2018	17.00	1.90	5	7.9%	17.00 [15.33; 18.67]
Yabuuchi	2021	58.50	6.10	4	7.7%	58.50 [52.52; 64.48]
Yamakage	2021	3.10	0.60	6	7.9%	3.10 [2.62; 3.58]
**Total (95% CI)**				**42**	**54.9%**	**18.69 [4.58; 32.81]**
Heterogeneity: $Tau^2 = 361.2999$; $Chi^2 = 1052.32$, df = 6 (P < 0.01); $I^2 = 99\%$						
**Total (95% CI)**				**81**	**100.0%**	**20.45 [10.67; 30.23]**
Heterogeneity: $Tau^2 = 315.9589$; $Chi^2 = 79440.66$, df = 12 (P = 0); $I^2 = 100\%$						
Test for subgroup differences: $Chi^2 = 0.14$, df = 1 (P = 0.70)						

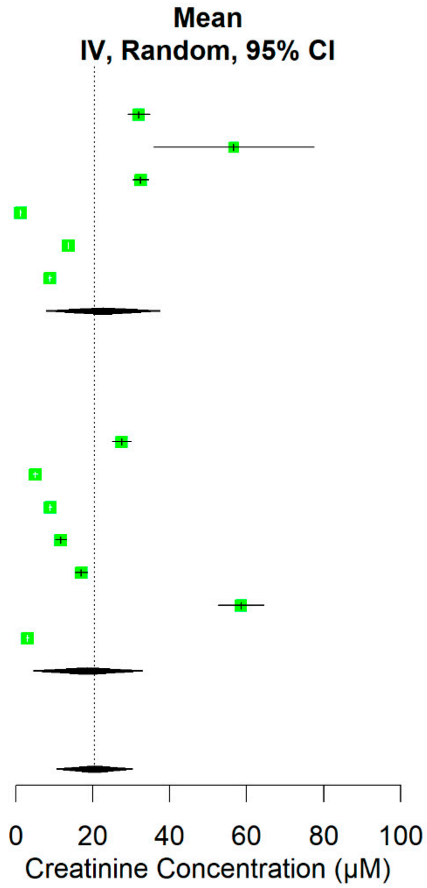

Study or Subgroup	Year	Mean	SD	Total	Weight	Mean IV, Random, 95% CI
Nephron loss						
Dou	2017	67.97	25.30	6	7.7%	67.97 [47.73; 88.21]
Hung	2016	86.00	47.40	9	7.1%	86.00 [55.03; 116.97]
Makhloufi	2020	55.60	5.90	8	8.1%	55.60 [51.51; 59.69]
Ni	2014	9.31	0.33	6	8.1%	9.31 [9.05; 9.57]
Tungasanga	2022	79.60	35.70	10	7.6%	79.60 [57.47; 101.73]
Yamada	2019	27.30	4.70	5	8.1%	27.30 [23.18; 31.42]
Total (95% CI)				44	46.6%	52.15 [27.81; 76.48]
Heterogeneity: Tau^2 = 851.4017; Chi^2 = 655.61, df = 5 (P < 0.01); I^2 = 99%						
Tubular damage						
Makhloufi	2020	81.00	12.45	8	8.0%	81.00 [72.37; 89.63]
Mishima	2017	16.70	3.50	6	8.1%	16.70 [13.90; 19.50]
Mishima	2018	28.10	4.20	8	8.1%	28.10 [25.19; 31.01]
Nanto-Hara	2020	31.20	1.04	5	8.1%	31.20 [30.29; 32.11]
Shiba	2018	50.50	16.30	6	7.9%	50.50 [37.46; 63.54]
Yabuuchi	2021	233.80	92.60	8	5.1%	233.80 [169.63; 297.97]
Yamakage	2021	47.80	3.80	7	8.1%	47.80 [44.98; 50.62]
Total (95% CI)				48	53.4%	64.81 [17.03; 112.60]
Heterogeneity: Tau^2 = 4025.9175; Chi^2 = 414.41, df = 6 (P < 0.01); I^2 = 99%						
**Total (95% CI)**				**92**	**100.0%**	**57.18 [33.53; 80.83]**
Heterogeneity: Tau^2 = 1796.1776; Chi^2 = 3751.86, df = 12 (P = 0); I^2 = 100%						
Test for subgroup differences: Chi^2 = 0.21, df = 1 (P = 0.64)						

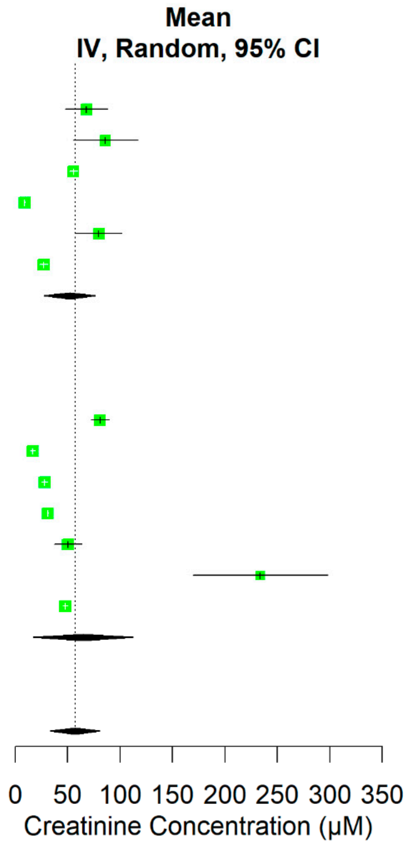

Figure S4. One-sided forest plots of creatinine concentrations (in μM) in healthy (top) and diseased (bottom) mice stratified by damage type.

Study or Subgroup	Year	Mean	SD	Total	Weight	Mean IV, Random, 95% CI
**Nephron loss**						
Dou	2017	8.75	0.69	6	7.2%	8.75 [8.20; 9.30]
Hung	2016	9.10	1.40	9	7.1%	9.10 [8.19; 10.01]
Ito	2012	14.64	1.79	10	7.1%	14.64 [13.53; 15.75]
Makhloufi	2020	3.30	0.30	8	7.2%	3.30 [3.09; 3.51]
Nakada	2019	9.50	1.30	7	7.1%	9.50 [8.54; 10.46]
Ni	2014	3.10	0.30	6	7.2%	3.10 [2.86; 3.34]
Nishikawa	2015	12.50	0.70	12	7.2%	12.50 [12.10; 12.90]
**Total (95% CI)**				**58**	**50.1%**	**8.68 [5.49; 11.87]**
Heterogeneity: $Tau^2 = 18.4094$; $Chi^2 = 2501.33$, df = 6 (P = 0); $I^2 = 100\%$						
**Tubular damage**						
Makhloufi	2020	2.82	0.20	8	7.2%	2.82 [2.68; 2.96]
Mishima	2017	8.50	1.40	4	7.0%	8.50 [7.13; 9.87]
Mishima	2018	10.30	1.20	6	7.1%	10.30 [9.34; 11.26]
Nanto-Hara	2020	9.20	1.00	9	7.2%	9.20 [8.55; 9.85]
Shiba	2018	9.10	1.10	5	7.1%	9.10 [8.14; 10.06]
Yabuuchi	2021	9.10	0.96	4	7.1%	9.10 [8.16; 10.04]
Yamakage	2021	15.50	0.96	6	7.2%	15.50 [14.73; 16.27]
**Total (95% CI)**				**42**	**49.9%**	**9.21 [6.44; 11.97]**
Heterogeneity: $Tau^2 = 13.7288$; $Chi^2 = 1804.38$, df = 6 (P = 0); $I^2 = 100\%$						
**Total (95% CI)**				**100**	**100.0%**	**8.94 [6.91; 10.98]**

Heterogeneity: $Tau^2 = 14.9055$; $Chi^2 = 4476.63$, df = 13 (P = 0); $I^2 = 100\%$
Test for subgroup differences: $Chi^2 = 0.06$, df = 1 (P = 0.81)

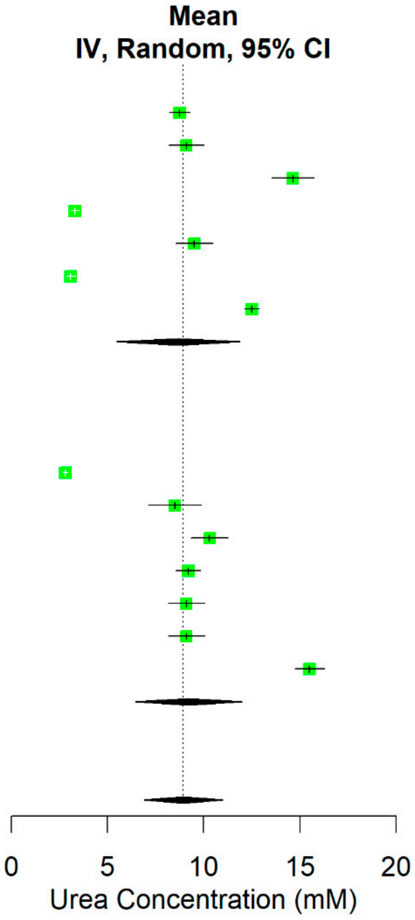

Study or Subgroup	Year	Mean	SD	Total	Weight	Mean IV, Random, 95% CI
**Nephron loss**						
Dou	2017	25.02	3.10	6	7.2%	25.02 [22.54; 27.50]
Hung	2016	22.40	7.00	9	7.2%	22.40 [17.83; 26.97]
Ito	2012	25.71	3.90	10	7.3%	25.71 [23.29; 28.13]
Makhloufi	2020	8.60	2.50	8	7.3%	8.60 [6.87; 10.33]
Nakada	2019	15.90	1.98	8	7.3%	15.90 [14.53; 17.27]
Ni	2014	5.90	0.40	6	7.3%	5.90 [5.58; 6.22]
Nishikawa	2015	24.30	15.00	12	7.0%	24.30 [15.81; 32.79]
**Total (95% CI)**				**59**	**50.5%**	**17.99 [11.76; 24.22]**
Heterogeneity: $Tau^2 = 67.0922$; $Chi^2 = 701.78$, df = 6 ($P < 0.01$); $I^2 = 99\%$						
**Tubular damage**						
Makhloufi	2020	11.70	2.52	8	7.3%	11.70 [9.95; 13.45]
Mishima	2017	19.50	7.00	6	7.2%	19.50 [13.90; 25.10]
Mishima	2018	22.50	6.50	8	7.2%	22.50 [18.00; 27.00]
Nanto-Hara	2020	29.50	3.10	5	7.2%	29.50 [26.78; 32.22]
Shiba	2018	47.70	20.30	6	6.4%	47.70 [31.46; 63.94]
Yabuuchi	2021	80.00	13.00	8	7.0%	80.00 [70.99; 89.01]
Yamakage	2021	77.30	5.10	7	7.2%	77.30 [73.52; 81.08]
**Total (95% CI)**				**48**	**49.5%**	**40.98 [20.15; 61.81]**
Heterogeneity: $Tau^2 = 775.3630$; $Chi^2 = 1124.24$, df = 6 ($P < 0.01$); $I^2 = 99\%$						
**Total (95% CI)**				**107**	**100.0%**	**29.44 [17.41; 41.46]**
Heterogeneity: $Tau^2 = 517.7458$; $Chi^2 = 2606.14$, df = 13 ($P = 0$); $I^2 = 100\%$						
Test for subgroup differences: $Chi^2 = 4.30$, df = 1 ($P = 0.04$)						

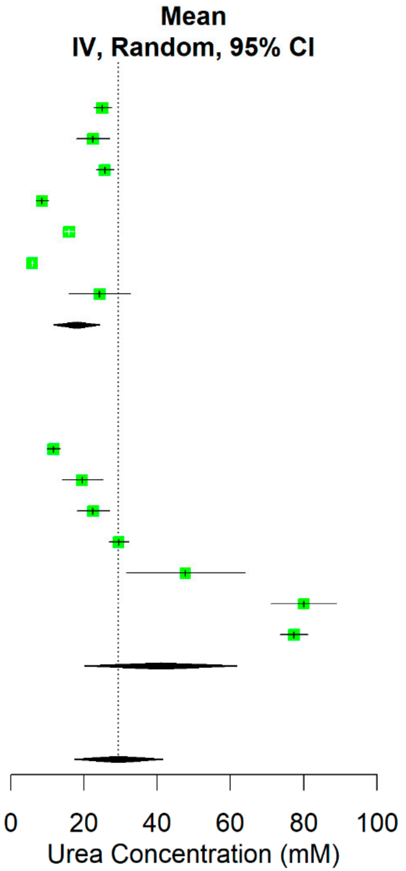

Figure S5. One-sided Forest plots of urea concentrations (mM) in healthy (top) and diseased (bottom) mice stratified by damage type.

Study or Subgroup	Year	Total	Disease Mean	SD	Total	Healthy Mean	SD	Ratio
Nephron loss								
Aoki	2015	8	40.52	31.50	8	7.09	2.23	5.72
Aoyama	2002	14	59.09	9.85	14	5.16	0.94	11.45
Aoyama	2003	5	60.97	31.46	4	9.38	9.38	6.50
Bolati	2012	8	24.39	21.20	9	3.75	0.99	6.50
Enomoto	2002	7	55.30	19.84	3	12.70	0.81	4.35
Iwasaki	2013	9	10.79	3.75	6	0.47	0.00	23.01
Jerez-Morales	2021	5	2.47	0.77	5	1.78	0.48	1.39
Kikuchi	2010	16	15.95	0.20	8	2.81	1.33	5.68
Kikuchi	2017	7	23.69	7.10	5	3.26	0.53	7.27
Kuwahara	2014	7	28.14	11.26	6	4.69	1.41	6.00
Lekawanvijit	2012	17	38.93	15.46	12	8.44	3.26	4.61
Lin	2020	5	41.74	21.11	4	5.63	1.41	7.41
Ling Lau	2019	4	10.57	3.08	5	2.79	0.72	3.79
Miyazaki	2000	5	66.13	25.33	4	7.50	0.94	8.82
Owada	2007	10	77.39	32.60	24	11.73	6.90	6.60
Shi	2018	6	46.81	9.05	6	8.72	0.33	5.37
Shimoishi	2007	7	17.40	2.12	7	1.20	0.56	14.50
Tumur	2008	6	36.58	25.30	6	2.81	1.15	13.02
Zeng	2016	9	19.36	2.50	13	6.91	1.62	2.80
Total (fixed effect, 95% CI)		155			149			
Total (random effects, 95% CI)								
Heterogeneity: $Tau^2 = 286.0887$; $Chi^2 = 1095.16$, df = 18 ($P < 0.01$); $I^2 = 98\%$								
Tubular damage								
Al Za'abi	2021	6	34.80	4.12	6	4.20	0.73	8.29
Al Za'abi	2021	6	132.60	12.20	6	14.40	2.30	9.21
Al Za'abi	2020	6	54.03	6.88	6	27.02	5.51	2.00
Ali	2018	6	20.30	4.90	6	2.90	1.96	7.00
Ali	2014	6	159.55	32.97	6	1.79	1.98	89.13
Ali	2019	6	62.85	19.50	6	2.35	2.30	26.74
Inami	2014	6	191.45	114.11	6	7.55	3.00	25.36
Iwata	2007	6	183.30	140.50	6	7.33	2.25	25.01
Kucey	2019	12	190.10	88.98	9	6.00	4.30	31.68
Kusumoto	2011	3	128.09	23.60	3	7.87	1.12	16.28
Matsushima	2017	3	151.90	51.30	3	4.02	1.63	37.79
Morisaki	2008	11	156.30	48.80	11	12.50	1.60	12.50
Sueyoshi	2019	11	183.60	68.50	12	6.88	0.01	26.69
Suleimani	2015	6	68.20	12.00	8	1.67	2.90	40.84
Won	2016	6	200.80	62.10	6	7.20	2.40	27.89
Total (fixed effect, 95% CI)		100			100			
Total (random effects, 95% CI)								
Heterogeneity: $Tau^2 = 3725.1825$; $Chi^2 = 720.96$, df = 14 ($P < 0.01$); $I^2 = 98\%$								
**Total (fixed effect, 95% CI)**		**255**			**249**			
**Total (random effects, 95% CI)**								
Heterogeneity: $Tau^2 = 3065.8980$; $Chi^2 = 2391.84$, df = 33 ($P = 0$); $I^2 = 99\%$								

Figure S6. The fold increase (the ratio of the mean uremic concentration to the healthy concentration) of plasma indoxyl sulfate in rats stratified by damage type.

Study or Subgroup	Year	Disease Total	Disease Mean	Disease SD	Healthy Total	Healthy Mean	Healthy SD	Ratio
Nephron loss								
Dou	2017	6	123.30	85.00	6	31.60	19.90	3.90
Hung	2016	9	37.10	3.80	9	12.79	2.20	2.90
Ito	2012	10	30.49	9.85	10	14.54	5.16	2.10
Makhloufi	2020	8	75.40	84.30	8	12.90	5.90	5.84
Nakada	2019	8	15.48	3.99	7	7.97	2.50	1.94
Nishikawa	2015	12	49.00	5.36	12	9.99	1.29	4.90
Tungasanga	2022	10	164.30	7.10	5	6.67	2.70	24.63
Yamada	2019	5	49.60	9.58	5	8.72	0.67	5.69
Total (fixed effect, 95% CI)		68			62			
Total (random effects, 95% CI)								
Heterogeneity: $Tau^2 = 2512.2897$; $Chi^2 = 2655.1$, df = 7 (P = 0); $I^2 = 100\%$								
Tubular damage								
Makhloufi	2020	8	161.80	93.60	8	14.50	5.66	11.16
Mishima	2017	6	20.20	5.80	4	3.68	0.86	5.49
Mishima	2018	8	86.80	17.50	6	16.90	3.00	5.14
Nanto-Hara	2020	5	79.30	15.40	9	4.03	3.50	19.68
Shiba	2018	6	10.68	13.10	5	1.48	0.01	7.22
Yabuuchi	2021	8	48.40	16.30	4	2.20	1.10	22.00
Yamakage	2021	7	49.30	5.30	6	14.67	1.13	3.36
Total (fixed effect, 95% CI)		48			42			
Total (random effects, 95% CI)								
Heterogeneity: $Tau^2 = 1188.7496$; $Chi^2 = 153.34$, df = 6 ($P < 0.01$); $I^2 = 96\%$								
**Total (fixed effect, 95% CI)**		**116**			**104**			
**Total (random effects, 95% CI)**								
Heterogeneity: $Tau^2 = 1850.3934$; $Chi^2 = 2827.97$, df = 14 (P = 0); $I^2 = 100\%$								

Figure S7. The fold change (the ratio of the mean uremic concentration to the healthy concentration) of plasma indoxyl sulfate in mice stratified by damage type.

Study or Subgroup	Year	Disease Total	Disease Mean	Disease SD	Healthy Total	Healthy Mean	Healthy SD	Ratio
Nephron loss								
Aoki	2015	8	93.70	25.00	8	30.06	12.50	3.12
Aoyama	2002	14	154.70	26.52	14	39.78	0.00	3.89
Aoyama	2003	5	159.12	59.31	4	35.36	0.00	4.50
Bolati	2012	8	124.64	57.50	9	35.36	0.27	3.52
Deguchi	2005	15	167.08	34.20	15	67.01	8.21	2.49
Enomoto	2002	7	159.12	23.40	3	35.36	15.31	4.50
Huang	2018	6	140.70	27.50	6	32.00	4.50	4.40
Iwasaki	2013	9	97.24	26.52	6	44.20	0.00	2.20
Jerez-Morales	2021	5	48.30	14.50	5	17.72	5.79	2.73
Kikuchi	2017	7	170.62	27.40	5	62.77	5.30	2.72
Kobayashi	2002	9	132.60	26.52	9	88.40	0.00	1.50
Kuwahara	2014	7	118.46	57.46	6	25.64	2.65	4.62
Lekawanvijit	2012	17	115.20	49.60	12	40.25	13.79	2.86
Lin	2020	5	114.92	17.68	4	35.36	0.00	3.25
Miyazaki	2000	5	132.60	17.38	4	44.20	8.84	3.00
Owada	2007	10	137.02	120.20	24	47.74	8.67	2.87
Shi	2018	6	176.80	12.40	6	38.90	2.65	4.54
Shimoishi	2007	7	173.27	35.08	7	33.59	32.80	5.16
Tóthová	2015	15	197.70	282.60	15	57.84	26.20	3.42
Tumur	2008	6	161.78	82.30	6	40.66	6.49	3.98
Watanabe	2013	11	113.15	21.22	4	57.46	11.49	1.97
Zeng	2016	9	85.75	10.61	13	35.36	5.30	2.43
Total (fixed effect, 95% CI)		191			185			
Total (random effects, 95% CI)								
Heterogeneity: $Tau^2 = 932.7477$; $Chi^2 = 343.37$, df = 21 ($P < 0.01$); $I^2 = 94\%$								
Tubular damage								
Al Za'abi	2021	6	58.20	12.50	6	20.50	3.90	2.84
Al Za'abi	2021	6	57.82	9.41	6	17.45	2.55	3.31
Al Za'abi	2020	6	141.20	47.08	6	20.98	3.92	6.73
Ali	2018	6	67.10	9.10	6	13.20	0.73	5.08
Ali	2014	6	205.57	30.50	6	63.09	11.70	3.26
Ali	2019	6	83.30	14.50	6	17.10	2.57	4.87
Inami	2014	6	138.79	49.50	6	29.17	2.30	4.76
Iwata	2007	6	171.10	23.80	6	16.80	2.10	10.18
Kucey	2019	12	207.90	71.30	9	21.89	5.05	9.50
Kusumoto	2011	3	99.90	17.10	3	18.40	1.80	5.43
Matsushima	2017	3	197.10	62.70	3	29.17	12.25	6.76
Morisaki	2008	11	130.83	43.98	11	17.68	2.92	7.40
Sueyoshi	2019	11	190.00	75.90	12	24.50	0.01	7.76
Suleimani	2015	6	136.80	36.80	8	80.60	19.10	1.70
Won	2016	6	76.40	8.37	6	21.97	3.14	3.48
Total (fixed effect, 95% CI)		100			100			
Total (random effects, 95% CI)								
Heterogeneity: $Tau^2 = 2290.1506$; $Chi^2 = 283.02$, df = 14 ($P < 0.01$); $I^2 = 95\%$								
**Total (fixed effect, 95% CI)**		**291**			**285**			
**Total (random effects, 95% CI)**								
Heterogeneity: $Tau^2 = 1410.9081$; $Chi^2 = 681.95$, df = 36 ($P < 0.01$); $I^2 = 95\%$								

Figure S8. The fold change (the ratio of the mean uremic concentration to the healthy concentration) of plasma creatinine in rats stratified by damage type.

Study or Subgroup	Year	Disease Total	Disease Mean	Disease SD	Healthy Total	Healthy Mean	Healthy SD	Ratio
Nephron loss								
Dou	2017	6	67.97	25.30	6	31.90	3.60	2.13
Hung	2016	9	86.00	47.40	9	56.60	31.90	1.52
Makhloufi	2020	8	55.60	5.90	8	32.40	3.10	1.72
Ni	2014	6	9.31	0.33	6	1.31	0.11	7.11
Tungasanga	2022	10	79.60	35.70	5	13.70	0.01	5.81
Yamada	2019	5	27.30	4.70	5	8.84	0.45	3.09
Total (fixed effect, 95% CI)		44			39			
Total (random effects, 95% CI)								
Heterogeneity: $Tau^2 = 316.5236$; $Chi^2 = 100.27$, df = 5 ($P < 0.01$); $I^2 = 95\%$								
Tubular damage								
Makhloufi	2020	8	81.00	12.45	8	27.50	3.68	2.95
Mishima	2017	6	16.70	3.50	4	5.06	0.75	3.30
Mishima	2018	8	28.10	4.20	6	9.00	0.60	3.12
Nanto-Hara	2020	5	31.20	1.04	9	11.70	2.40	2.67
Shiba	2018	6	50.50	16.30	5	17.00	1.90	2.97
Yabuuchi	2021	8	233.80	92.60	4	58.50	6.10	4.00
Yamakage	2021	7	47.80	3.80	6	3.10	0.60	15.42
Total (fixed effect, 95% CI)		48			42			
Total (random effects, 95% CI)								
Heterogeneity: $Tau^2 = 1434.7340$; $Chi^2 = 369.63$, df = 6 ($P < 0.01$); $I^2 = 98\%$								
**Total (fixed effect, 95% CI)**		**92**			**81**			
**Total (random effects, 95% CI)**								
Heterogeneity: $Tau^2 = 459.7722$; $Chi^2 = 1048.69$, df = 12 ($P < 0.01$); $I^2 = 99\%$								

[illegible]

Figure S9. The fold change (the ratio of the mean uremic concentration to the healthy concentration) of plasma creatinine in mice stratified by damage type.

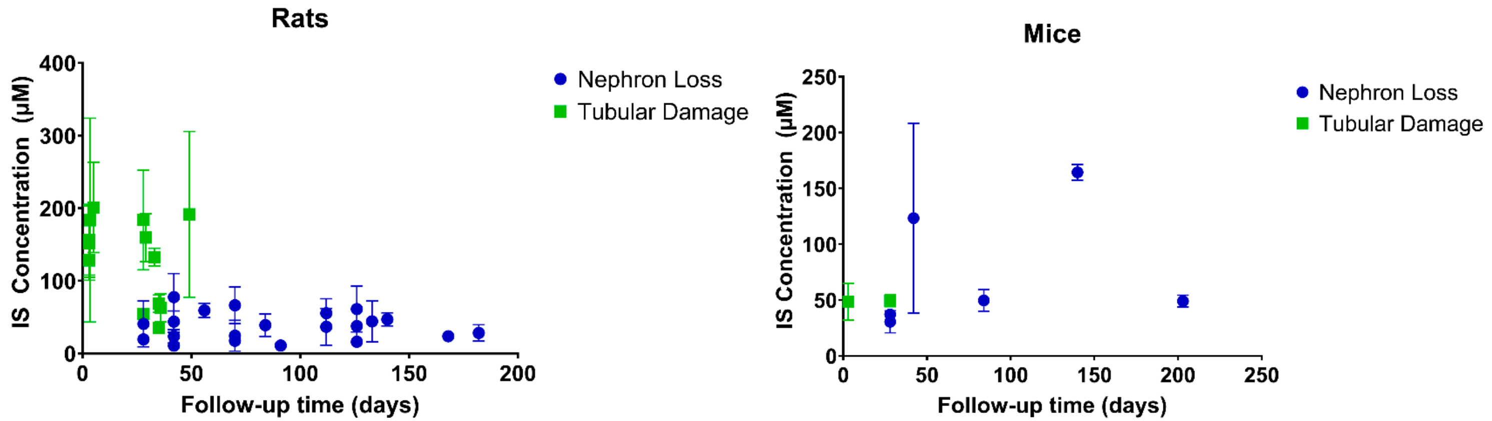

Figure S10. The concentrations of plasma IS in tubular injury and nephron loss models in rats (left) and mice (right) based on follow-up period (days).

Study or Subgroup	Year	Mean	SD	Total	Weight	Mean IV, Random, 95% CI
**Sprague-Dawley**						
Ali	2018	2.90	1.96	6	6.7%	2.90 [1.33; 4.47]
Inami	2014	7.55	3.00	6	6.6%	7.55 [5.15; 9.95]
Iwata	2007	7.33	2.25	6	6.7%	7.33 [5.53; 9.13]
Kusumoto	2011	7.87	1.12	3	6.8%	7.87 [6.60; 9.14]
Matsushima	2017	4.02	1.63	3	6.7%	4.02 [2.18; 5.86]
Morisaki	2008	12.50	1.60	11	6.8%	12.50 [11.55; 13.45]
Won	2016	7.20	2.40	6	6.7%	7.20 [5.28; 9.12]
**Total (95% CI)**				**41**	**47.0%**	**7.09 [4.73; 9.45]**
Heterogeneity: $Tau^2 = 9.3596$; $Chi^2 = 145.14$, df = 6 ($P < 0.01$); $I^2 = 96\%$						
**Wistar**						
Al Za'abi	2021	4.20	0.73	6	6.8%	4.20 [3.62; 4.78]
Al Za'abi	2021	14.40	2.30	6	6.7%	14.40 [12.56; 16.24]
Al Za'abi	2020	27.02	5.51	6	6.0%	27.02 [22.61; 31.43]
Ali	2014	1.79	1.98	6	6.7%	1.79 [0.21; 3.37]
Ali	2019	2.35	2.30	6	6.7%	2.35 [0.51; 4.19]
Kucey	2019	6.00	4.30	9	6.5%	6.00 [3.19; 8.81]
Sueyoshi	2019	6.88	0.01	12	6.9%	6.88 [6.87; 6.89]
Suleimani	2015	1.67	2.90	8	6.7%	1.67 [-0.34; 3.68]
**Total (95% CI)**				**59**	**53.0%**	**7.90 [2.05; 13.76]**
Heterogeneity: $Tau^2 = 70.0944$; $Chi^2 = 314.28$, df = 7 ($P < 0.01$); $I^2 = 98\%$						
**Total (95% CI)**				**100**	**100.0%**	**7.45 [4.34; 10.55]**

Heterogeneity: $Tau^2 = 36.6115$; $Chi^2 = 486.97$, df = 14 ($P < 0.01$); $I^2 = 97\%$
Test for subgroup differences: $Chi^2 = 0.06$, df = 1 ($P = 0.80$)

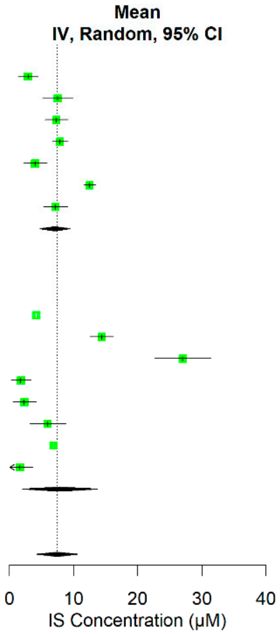

Study or Subgroup	Year	Mean	SD	Total	Weight	Mean IV, Random, 95% CI
**Sprague-Dawley**						
Ali	2018	20.30	4.90	6	7.5%	20.30 [16.38; 24.22]
Inami	2014	191.45	114.11	6	4.7%	191.45 [100.14; 282.76]
Iwata	2007	183.30	140.50	6	3.9%	183.30 [70.88; 295.72]
Kusumoto	2011	128.09	23.60	3	7.1%	128.09 [101.38; 154.80]
Matsushima	2017	151.90	51.30	3	6.0%	151.90 [93.85; 209.95]
Morisaki	2008	156.30	48.80	11	7.1%	156.30 [127.46; 185.14]
Won	2016	200.80	62.10	6	6.4%	200.80 [151.11; 250.49]
**Total (95% CI)**				**41**	**42.7%**	**140.52 [89.50; 191.54]**
Heterogeneity: $Tau^2 = 3904.5268$; $Chi^2 = 228.15$, df = 6 ($P < 0.01$); $I^2 = 97\%$						
**Wistar**						
Al Za'abi	2021	34.80	4.12	6	7.5%	34.80 [31.50; 38.10]
Al Za'abi	2021	132.60	12.20	6	7.4%	132.60 [122.84; 142.36]
Al Za'abi	2020	54.03	6.88	6	7.5%	54.03 [48.52; 59.54]
Ali	2014	159.55	32.97	6	7.1%	159.55 [133.17; 185.93]
Ali	2019	62.85	19.50	6	7.4%	62.85 [47.25; 78.45]
Kucey	2019	190.10	88.98	12	6.3%	190.10 [139.76; 240.44]
Sueyoshi	2019	183.60	68.50	11	6.7%	183.60 [143.12; 224.08]
Suleimani	2015	68.20	12.00	6	7.4%	68.20 [58.60; 77.80]
**Total (95% CI)**				**59**	**57.3%**	**107.95 [65.40; 150.50]**
Heterogeneity: $Tau^2 = 3609.3523$; $Chi^2 = 512.47$, df = 7 ($P < 0.01$); $I^2 = 99\%$						
**Total (95% CI)**				**100**	**100.0%**	**121.72 [89.29; 154.14]**
Heterogeneity: $Tau^2 = 3658.9303$; $Chi^2 = 844.82$, df = 14 ($P < 0.01$); $I^2 = 98\%$						
Test for subgroup differences: $Chi^2 = 0.92$, df = 1 ($P = 0.34$)						

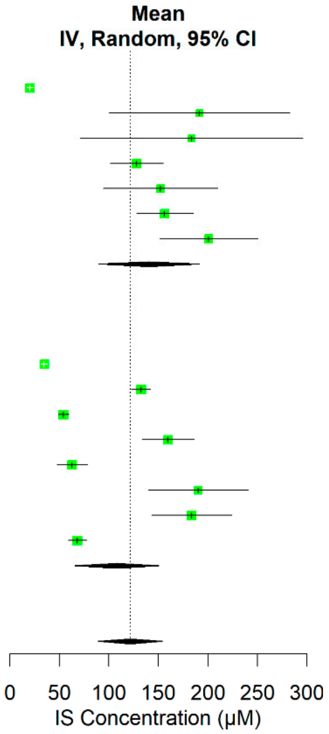

Figure S11. One-sided Forest plots of plasma indoxyl sulfate concentrations (IS; in μM) in healthy (top) and diseased (bottom) in tubular damage rat models stratified by strain type.

Study or Subgroup	Year	Mean	SD	Total	Weight	Mean IV, Random, 95% CI
Sprague-Dawley						
Ali	2018	4.40	0.73	6	10.6%	4.40 [3.82; 4.98]
Inami	2014	8.90	2.30	6	8.6%	8.90 [7.06; 10.74]
Iwata	2007	7.18	2.10	6	8.9%	7.18 [5.50; 8.86]
Kusumoto	2011	9.32	1.80	3	8.2%	9.32 [7.28; 11.36]
Matsushima	2017	.	12.25	3	0.0%	
Morisaki	2008	7.80	2.92	11	8.8%	7.80 [6.07; 9.53]
Won	2016	7.40	3.14	6	7.3%	7.40 [4.89; 9.91]
Total (95% CI)				41	52.4%	7.36 [5.79; 8.93]
Heterogeneity: Tau^2 = 3.0323; Chi^2 = 52.99, df = 5 (P < 0.01); I^2 = 91%						
Wistar						
Al Za'abi	2021	3.00	3.90	6	6.2%	3.00 [-0.12; 6.12]
Al Za'abi	2021	3.73	2.55	6	8.2%	3.73 [1.69; 5.77]
Al Za'abi	2020	6.13	3.92	6	6.2%	6.13 [2.99; 9.27]
Ali	2014	6.36	11.70	6	1.4%	6.36 [-3.00; 15.72]
Ali	2019	3.70	2.57	6	8.2%	3.70 [1.64; 5.76]
Kucey	2019	6.44	5.05	9	5.9%	6.44 [3.14; 9.74]
Sueyoshi	2019	7.05	0.01	12	10.8%	7.05 [7.04; 7.06]
Suleimani	2015	22.80	19.10	8	0.7%	22.80 [9.56; 36.04]
Total (95% CI)				59	47.6%	5.39 [3.86; 6.92]
Heterogeneity: Tau^2 = 2.4040; Chi^2 = 32.76, df = 7 (P < 0.01); I^2 = 79%						
**Total (95% CI)**				**100**	**100.0%**	**6.44 [5.25; 7.62]**
Heterogeneity: Tau^2 = 3.3718; Chi^2 = 121.29, df = 13 (P < 0.01); I^2 = 89%						
Test for subgroup differences: Chi^2 = 3.10, df = 1 (P = 0.08)						

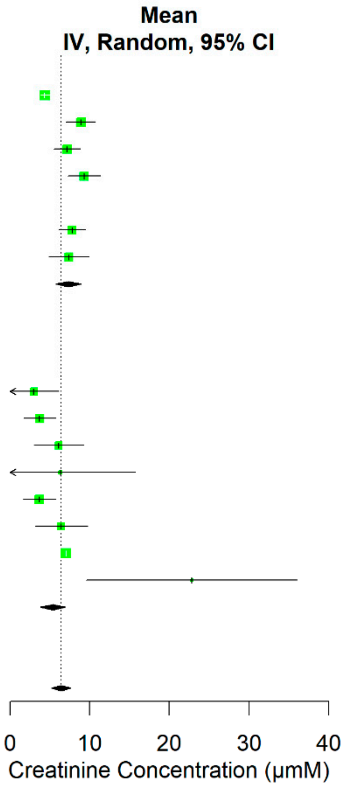

Study or Subgroup	Year	Mean	SD	Total	Weight	Mean IV, Random, 95% CI
Sprague-Dawley						
Ali	2018	14.90	9.10	6	8.1%	14.90 [7.62; 22.18]
Inami	2014	40.50	49.50	6	6.0%	40.50 [0.89; 80.11]
Iwata	2007	59.90	23.80	6	7.5%	59.90 [40.86; 78.94]
Kusumoto	2011	42.62	17.10	3	7.5%	42.62 [23.27; 61.97]
Matsushima	2017	.	62.70	3	0.0%	
Morisaki	2008	48.57	43.98	11	7.1%	48.57 [22.58; 74.56]
Won	2016	28.50	8.37	6	8.1%	28.50 [21.80; 35.20]
Total (95% CI)				41	44.3%	36.84 [22.22; 51.46]
Heterogeneity: Tau^2 = 237.2539; Chi^2 = 27.82, df = 5 (P < 0.01); I^2 = 82%						
Wistar						
Al Za'abi	2021	18.00	12.50	6	8.0%	18.00 [8.00; 28.00]
Al Za'abi	2021	16.41	9.41	6	8.1%	16.41 [8.88; 23.94]
Al Za'abi	2020	42.94	47.08	6	6.2%	42.94 [5.27; 80.61]
Ali	2014	150.71	30.50	6	7.2%	150.71 [126.31; 175.11]
Ali	2019	22.00	14.50	6	7.9%	22.00 [10.40; 33.60]
Kucey	2019	68.20	71.30	12	6.0%	68.20 [27.86; 108.54]
Sueyoshi	2019	45.99	75.90	11	5.6%	45.99 [1.14; 90.84]
Suleimani	2015	77.50	36.80	6	6.8%	77.50 [48.05; 106.95]
Total (95% CI)				59	55.7%	54.34 [22.11; 86.57]
Heterogeneity: Tau^2 = 1957.3946; Chi^2 = 126.81, df = 7 (P < 0.01); I^2 = 94%						
**Total (95% CI)**				**100**	**100.0%**	**47.02 [28.03; 66.01]**

Heterogeneity: Tau^2 = 1150.7611; Chi^2 = 154.65, df = 13 (P < 0.01); I^2 = 92%
Test for subgroup differences: Chi^2 = 0.94, df = 1 (P = 0.33)

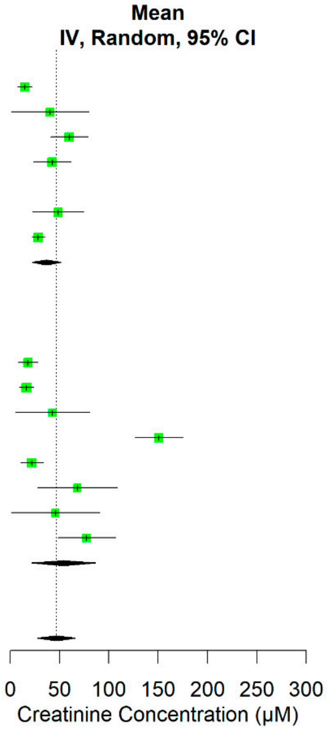

Figure S12. One-sided forest plots of plasma creatinine concentrations (Cr; in μM) in healthy (top) and diseased (bottom) in tubular damage rat models stratified by strain type.

Study or Subgroup	Year	Mean	SD	Total	Weight	Mean IV, Random, 95% CI
**Sprague-Dawley**						
Ali	2018	4.40	0.24	6	7.4%	4.40 [4.21; 4.59]
Inami	2014	8.90	1.39	6	7.3%	8.90 [7.79; 10.01]
Iwata	2007	7.18	2.62	6	7.0%	7.18 [5.08; 9.28]
Kusumoto	2011	9.32	2.13	3	6.9%	9.32 [6.91; 11.73]
Morisaki	2008	7.80	1.18	11	7.4%	7.80 [7.10; 8.50]
Won	2016	7.40	0.94	6	7.4%	7.40 [6.65; 8.15]
**Total (95% CI)**				**38**	**43.3%**	**7.37 [5.90; 8.84]**
Heterogeneity: Tau^2 = 2.9063; Chi^2 = 201.86, df = 5 ($P < 0.01$); I^2 = 98%						
**Wistar**						
Al Za'abi	2021	3.00	1.50	6	7.3%	3.00 [1.80; 4.20]
Al Za'abi	2021	3.73	0.61	6	7.4%	3.73 [3.24; 4.22]
Al Za'abi	2020	6.13	3.77	6	6.6%	6.13 [3.11; 9.15]
Ali	2014	6.36	1.96	6	7.2%	6.36 [4.79; 7.93]
Ali	2019	3.70	0.49	6	7.4%	3.70 [3.31; 4.09]
Kucey	2019	6.44	2.14	9	7.2%	6.44 [5.04; 7.84]
Sueyoshi	2019	7.05	0.01	12	7.4%	7.05 [7.04; 7.06]
Suleimani	2015	22.80	5.10	8	6.3%	22.80 [19.27; 26.33]
**Total (95% CI)**				**59**	**56.7%**	**7.26 [3.03; 11.49]**
Heterogeneity: Tau^2 = 36.4257; Chi^2 = 579.87, df = 7 ($P < 0.01$); I^2 = 99%						
**Total (95% CI)**				**97**	**100.0%**	**7.29 [4.98; 9.60]**
Heterogeneity: Tau^2 = 18.7727; Chi^2 = 1329.51, df = 13 ($P < 0.01$); I^2 = 99%						
Test for subgroup differences: Chi^2 = 0.00, df = 1 ($P = 0.96$)						

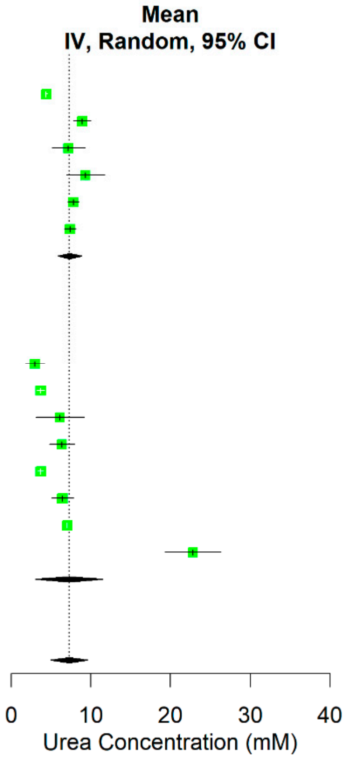

Study or Subgroup	Year	Mean	SD	Total	Weight	Mean IV, Random, 95% CI
**Sprague-Dawley**						
Ali	2018	20.30	4.90	6	7.5%	20.30 [16.38; 24.22]
Inami	2014	191.45	114.11	6	4.7%	191.45 [100.14; 282.76]
Iwata	2007	183.30	140.50	6	3.9%	183.30 [70.88; 295.72]
Kusumoto	2011	128.09	23.60	3	7.1%	128.09 [101.38; 154.80]
Matsushima	2017	151.90	51.30	3	6.0%	151.90 [93.85; 209.95]
Morisaki	2008	156.30	48.80	11	7.1%	156.30 [127.46; 185.14]
Won	2016	200.80	62.10	6	6.4%	200.80 [151.11; 250.49]
**Total (95% CI)**				**41**	**42.7%**	**140.52 [89.50; 191.54]**
Heterogeneity: Tau^2 = 3904.5268; Chi^2 = 228.15, df = 6 ($P < 0.01$); I^2 = 97%						
**Wistar**						
Al Za'abi	2021	34.80	4.12	6	7.5%	34.80 [31.50; 38.10]
Al Za'abi	2021	132.60	12.20	6	7.4%	132.60 [122.84; 142.36]
Al Za'abi	2020	54.03	6.88	6	7.5%	54.03 [48.52; 59.54]
Ali	2014	159.55	32.97	6	7.1%	159.55 [133.17; 185.93]
Ali	2019	62.85	19.50	6	7.4%	62.85 [47.25; 78.45]
Kucey	2019	190.10	88.98	12	6.3%	190.10 [139.76; 240.44]
Sueyoshi	2019	183.60	68.50	11	6.7%	183.60 [143.12; 224.08]
Suleimani	2015	68.20	12.00	6	7.4%	68.20 [58.60; 77.80]
**Total (95% CI)**				**59**	**57.3%**	**107.95 [65.40; 150.50]**
Heterogeneity: Tau^2 = 3609.3523; Chi^2 = 512.47, df = 7 ($P < 0.01$); I^2 = 99%						
**Total (95% CI)**				**100**	**100.0%**	**121.72 [89.29; 154.14]**
Heterogeneity: Tau^2 = 3658.9303; Chi^2 = 844.82, df = 14 ($P < 0.01$); I^2 = 98%						
Test for subgroup differences: Chi^2 = 0.92, df = 1 ($P = 0.34$)						

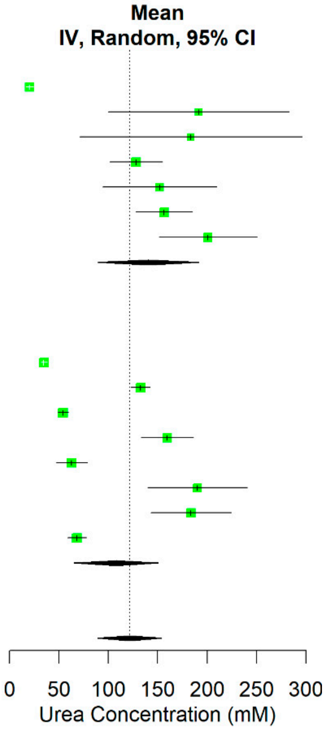

Figure S13. One-sided forest plots of plasma urea concentrations (mM) in healthy (left) and diseased (right) in tubular damage rat models stratified by strain type.

Tables

Supplementary Table S1. Overview of PBUTs concentration in animals						
**Rat**						
Strain	Disease model	Sample	Biomarker levels/clearance (uremic toxins and conventional markers) Healthy/Sham	Biomarker levels/clearance (uremic toxins and conventional markers) in disease models	Animal number	Ref
Sprague-Dawley	CKD (5/6 nephrectomy)	Serum		IS: 23.45±9.2 μM[a]<br>Cr: 139.3 ± 35.4 μM[ab]<br>Urea: 28.1 ± 7.0 mM[ab]<br>ClCr: 0.50 ± 0.24 mL/min[b]<br>6 weeks	n=12<br>(n=6 for IS)	Niwa T. et al., 1994 [71]
		Urine		IS: 2.2±0.25 mg/day<br>6 weeks		
		Serum	IS: 7.5±0.938 μM[a]<br>Cr: 44.20±8.84 μM[a]<br>Urea: 7.14 ± 0.71 mM[a]	IS: 66.13±25.33 μM[a]<br>Cr: 132.60±17.68 μM[a]<br>Urea: 24.64 ± 2.85 mM[a]<br>10 weeks	Control (n=4)<br>CRF (n=5)	Miyazaki T. et al., 2000 [72]*
		Urine	IS: 3.35±0.81 mg/day<br>Cr: 2.15±0.23 ml/min	IS: 3.36±1.17 mg/day<br>Cr: 0.65±0.12 ml/min<br>10 weeks		
		Serum	IS: 12.7 ± 0.814 μM<br>Cr: 35.36±15.31 μM[a]<br>Urea: 8.21 ± 0.62 mM[a]	IS: 55.3±19.84 μM<br>Cr: 159.12±23.4 μM[a]<br>Urea: 36.07 ± 4.72 mM[a]<br>16 weeks	Control (n=3)<br>CRF (n=7)	Enomoto A. et al., 2002 [73]*
		Urine	IS: 3.01 ± 0.85 mg/day<br>ClCr: 2.59 ± 0.121 ml/min	IS: 3.9±1.16 mg/day<br>ClCr: 0.76±0.185 ml/min		
		Plasma	IS: 9.38±9.38 μM[a]<br>Cr: 35.36±0.00 μM[a]<br>Urea: 8.93 ± 2.14 mM[a]	IS: 60.97±31.46 μM[a]<br>Cr: 159.12±59.31 μM[a]<br>Urea: 33.57 ± 9.58 mM[a]<br>18 weeks	Normal (n=4)<br>CRF (n=5)	Aoyama I. et al., 2003 [74]*

		Urine	IS: 4.1±3.2 mg/day<br>Cr: 3.08±0.74 ml/min	IS: 4.1±1.12 mg/day<br>Cr: 0.72±0.268 ml/min<br>18 weeks		
		Serum		IS:37.6±23.2 μM<br>HA: 11.6±4.8 μM<br>IAA: 1.0±0.57 μM<br>ClCr: 0.226 ± 0.048 mL/min/100g<br>18 weeks	n=8	Satoh M. et al., 2003 [75]
		Serum	IS: 2.81 ± 1.33 μM[a]<br>HA: 10.05 ± 2.21 μM[a]<br>pCS: 0.11± 0.00 μM[a]<br>PS: 0.041 ± 0.011 Mg/dl	IS: 15.95 ± 0.2 μM[a]<br>HA: 38.51 ± 23.68 μM[a]<br>pCS: 1.01 ± 1.28 μM[a]<br>PS: 0.146 ± 0.092 mg/dl	Normal (n=8)<br>CRF (n=16)	Kikuchi K. et al., 2010 [76]*
		Urine	IS: 88.64 ± 17.25 μM[a]<br>HA: 1780.43 ± 205.23 μM[a]<br>pCS: 4.73 ± 2.55 μM[a]<br>PS: 91.82 ± 9.81 μM[a]	IS: 124.29 ± 41.28 μM[a]<br>HA: 1802.76 ± 200.92 μM[a]<br>pCS: 6.85 ± 4.04 μM[a]<br>PS: 88.93 ± 20.8 μM[a]<br>18 weeks		
		Serum	IS: 8.44±3.26 μM[a]<br>Cr: 40.25±13.79 μM[a]<br>ClCr: 3.47 ± 1.178 mL/min/kg	IS: 38.93±15.46 μM[a]<br>Cr: 115.20±49.6 μM[a]<br>ClCr: 1.19 ±0.91 mL/min/kg	Sham (n=12)<br>CRF (n=17)	Lekawanvijit S. et al., 2012 [77]*
		Urine	IS: 1420.66±350.95 μM[a]<br>GFR 10.93 ± 1.212 mL/min/kg	IS: 523.9±150.82 μM[a]<br>GFR 0.93 ± 0.91 mL/min/kg<br>12 weeks		
		Serum	pCS: 0.55± 0.51 μM<br>Cr: 57.46±11.49 μM[a]<br>Urea: 6.7 ± 0.7 mM[a]<br>ClCr: 2.46 ± 0.96 mL/min	pCS: 2.04± 2.22 μM<br>Cr: 113.15±21.22 μM[a]<br>Urea: 16.7 ± 4.0 mM[a]<br>ClCr: 0.66 ± 0.12 mL/min<br>16 weeks, Cr and BUN	Sham (n=4)<br>CKD (n=11)	Watanabe H. et al., 2013 [13]*
		Serum	IS: 7.09 ± 2.23 μM[ab]<br>Cr: 30.06±12.5 μM[a]<br>Urea: 6.3 ± 0.81 mM[a]<br>ClCr: 3.10 ± 1.13 mL/min	IS: 40.52 ± 31.5 μM[ab]<br>Cr: 93.70±25.0 μM[a]<br>Urea: 25.3 ± 7.7 mM[a]	Sham (n=8)<br>5/6th (n=8)	Aoki K. et al., 2015 [78]*

				ClCr: 0.88 ± 0.25 mL/min<br>4 weeks		
		Serum	IS: 3.26 ± 0.53 μM[ab]<br>HA: 13.2 ± 3.0 μM[ab]<br>pCS: 0.30 ± 0.067 μM[ab]<br>Cr : 62.77±5.3 μM[a]<br>ClCr: 2.99 ± 0.32 mL/min	IS: 23.69 ± 7.1 μM[ab]<br>HA: 59.2 ± 14.2 μM[ab]<br>pCS: 4.72 ± 2.4 μM[ab]<br>Cr: 170.62±27.40 μM[a]<br>ClCr: 1.11 ± 0.16 mL/min<br>Week 24	Normal (n=5)<br>CKD (n=7)	Kikuchi M. et al., 2017 [79]*
		Urine	IS: 2.23 ± 0.37 mg/day[b]<br>HA: 22.1 ± 1.33 mg/day[b]<br>pCS: 0.39 ± 0.059 mg/day[b]	IS: 3.60 ± 0.39 mg/day[b]<br>HA: 24.43 ± 1.72 mg/day[b]<br>pCS: 0.716 ± 0.157 mg/day[b]		
	CKD (5/6 nephrectomy)	Plasma	IS: 2.79± 0.72 μM[a]<br>pCS: 0.60± 0.60 μM[a]<br>Urea: 7.18 ± 0.39 mM[a]<br>ClCr: 10.3 ± 4.47 mL/min/kg	IS: 10.57± 3.08 μM[a]<br>pCS: 2.39± 1.6 μM[a]<br>Urea: 57.5 ± 18.5 mM[a]<br>ClCr: 0.8 ± 0.2 mL/min/kg<br>Week 6	Normal (n=5)<br>CKD (n=4)	Ling Lau et al., 2018 [80]*
		Serum	IS: 6.91 ± 1.62 μM[a]<br>Cr: 35.36±5.30 μM[a]<br>Urea: 6.42 ± 4.82 mM[a]<br>ClCr: 6.09 ± 1.15 mL/min/kg	IS: 19.36 ± 2.5 μM[a]<br>Cr: 85.75±10.61 μM[a]<br>Urea: 40.71 ± 9.2 mM[a]<br>ClCr: 2.45 ± 1.23 mL/min/kg<br>4 weeks	Control (n=13)<br>CKD (n=9)	Zeng et al., 2016 [81]*
		Serum	IS: 5.63±1.41 μM[a]<br>pCS: 4.78±4.25 μM[a]<br>Cr: 35.36±0.0 μM[a]<br>Urea: 4.18 ± 0.43 mM[a]	IS: 41.74±21.11μM[a]<br>pCS: 57.92 ± 23.9 μM[a]<br>Cr: 114.92±17.68 μM[a]<br>Urea: 22.4 ± 4.86 mM[a]	Normal (n=4)<br>CKD (n=5)	Lin et al., 2020 [82]*
		Serum	IS: 3.75±0.99 μM[a]<br>Cr: 35.36±0.27 μM[a]<br>ClCr: 3.68 ± 0.13 mL/min	IS: 24.39±21.2 μM[a]<br>Cr: 124.64±57.5 μM[a]<br>ClCr: 1.14 ± 0.16 mL/min<br>10 weeks	Normal (n=9)<br>CKD (n=8)	Bolati D et al., 2013 [83]*
		Serum	IS: 0.469± 0.00 μM[a]<br>Cr: 44.20±0.0 μM[a]<br>Urea: 6.5 ± 0.36 mM[a]	IS: 10.79± 3.75 μM[a]<br>Cr: 97.24±26.52 μM[a]<br>Urea: 15.78 ± 3.96 mM[a]	Control (n=6)<br>CKD (n=9)	Iwasaki Y.et al., 2013 [84]*

			ClCr: 2.8 ± 0.2 mL/min	ClCr: 1.1 ± 0.3 mL/min<br>13 weeks		
		Serum	IS: 2.81±1.15 μM[a]<br>Cr: 40.66±6.49 μM[a]<br>Urea: 6.07 ± 0.87 mM[a]	IS: 36.58±25.3 μM[a]<br>Cr: 161.78±82.3 μM[a]<br>Urea: 30.85 ± 15.1 mM[a]<br>Week 16	Normal (n=6)<br>CKD (n=6)	Tumur Z. et al., 2008 [85]*
		Urine	IS: 3±0.98 mg/day<br>Cr: 3.404±0.181 ml/min	IS: 2.14±0.69 mg/day<br>Cr: 0.83 ± 0.49 ml/min		
		Serum	IS:4.69 ± 1.41 μM[a]<br>HA: 9.49 ± 3.35 μM[a]<br>pCS: 0.071 ± 0.028 μM[a]<br>Cr: 25.64 ± 2.65 μM[a]<br>Urea: 7.46 ± 0.2 mM[a]<br>ClCr: 4.48 ± 0.42 mL/min	IS: 28.14± 11.26 μM[a]<br>HA: 43.53 ± 18.42 μM[a]<br>pCS: 0.342 ± 0.191 μM[a]<br>Cr: 118.46 ± 57.46 μM[a]<br>Urea: 25.8 ± 6.2 mM[a]<br>ClCr: 1.28 ± 0.53 mL/min<br>Week 26	Control (n=6)<br>CKD (n=7)	Kuwahara M. et al., 2014 [86]*
		Plasma	Urea: 9.33 ± 0.15 mM[a]<br>Cr: 38.90 ± 2.65 μM[a]<br>pCS: 0.266 ± 0.05 μM[a]<br>IS: 8.72 ± 0.33 μM[a]<br>3-IAA: 0.17 ± 0.057 μM[a]<br>HA: below LOD	Urea: 54.6 ± 22.4 mM[a]<br>Cr: 176.8 ± 12.4 μM[a]<br>pCS: 33.8 ± 6.59 μM[a]<br>μM[a]<br>IS: 46.81 ± 9.05 μM[a]<br>3-IAA: 0.571 ± 0.11 μM[a]<br>HA: 7.2 ± 5.08 μM[a]<br>20 weeks	Both groups n=6	Shi Y. et al., 2018 [87]*
		Serum	IS: 4.41±0.98 μM[a]<br>Cr: 36.3± 0.9 μM	IS: 21.11 ±4.03 μM[a]<br>Cr: 106.2 ± 7.1 μM<br>12 weeks	Both groups n=6	Plam F. et al., 2010 [88]
		Plasma	Cr: 17.72 ± 5.79 μM[ab]<br>IS: 1.78 ± 0.48 μM[ab]	Cr: 48.3 ± 14.5 μM[ab]<br>IS: 2.47 ± 0.77 μM[ab]	Both groups n=5	Jerez-Morales et al., 2021 [89]*
	CKD (3/4 nephrectomy)	Serum	Cr: 88.4 ± 0.0 μM[a]<br>Urea: 24.3 ± 3.3 mM[a]<br>ClCr: 1.078 ± 0.081 mL/min/100g	IS: 44.09±28.14 μM[a]<br>Cr: 132.60±26.52 μM[a]<br>Urea: 32.1 ± 4.2 mM[a]<br>ClCr: 0.168 ± 0.03 mL/min/100g<br>Week 19	Pre (n=9)<br>Post (n=9)	Kobayashi N. et al., 2002 [90]

		Urine		IS: 4.52±1.08 mg/day		
	Unilateral nephrectomy (½ Nx)	Serum	IS: 5.16±0.938 μM[a]<br>Cr: 39.78±0.00 μM[a b]<br>Urea: 10.34 ± 0.357 mM[a]	IS: 59.09±9.85 μM[a]<br>Cr: 154.70±26.52 μM[ab]<br>Urea: 27.13 ± 0.714 mM[a]	Pre (n=14)<br>Post (n=14)	Aoyama I. et al., 2002 [91]*
		Urine	IS: 3.04±0.46 mg/day<br>Cr: 1.80±0.12 ml/min[b]	IS: 3.98±0.26 mg/day<br>Cr: 0.633 ± 0.06 ml/min[b]<br>8 weeks		
	0.75% Adenine (3 wks)<br>Cisplatin AKI 10 mg/kg	Plasma	Cr: 21.97 ± 3.14 μM[ab]<br>Urea: 7.4 ± 0.94 mM[ab]<br>IS: 7.2 ± 2.4 μM[ab]	Cr: 76.4 ± 8.37 μM[ab]<br>Urea: 28.5 ± 1.25 mM[ab]<br>IS: 200.8 ± 62.1 μM[ab]<br>5 days	Both groups n=6	Won AJ. et al., 2016 [92]*
		Urine	Cr: 0.27 ± 0.14 md/gL<br>BUN: 1537 ± 165 mg/dL	Cr: 0.45 ± 0.14 mg/dL<br>BUN: 357 ± 28		
	Remnant kidney model<br>Adenine induced CKD/uremia	Plasma	IS: 2.9±1.96 μM<br>Cr: 13.2±0.73 μM<br>Urea 4.4 ± 0.24 mM	IS 20.3± 4.90 μM<br>Cr: 67.1±9.1 μM<br>Urea 14.9 ± 1.47 mM	Both groups n=6	Ali BH. et al., 2018 [93]*
	Adenine induced uraemia	Serum	IS: 7.55±3 μM[a]<br>Cr: 29.17±2.30 μM[a]<br>Urea: 8.9 ± 1.39 mM[a]	IS: 191.45±114.11 μM[a]<br>Cr: 138.79±49.50 μM[a]<br>Urea: 40.5 ± 7.8 mM[a]<br>7 weeks S	Both groups n=6	Inami Y. et al., 2014 [94]*
	Cisplatin induced	Serum	IS: 7.33 ± 2.25 μM[ab]<br>Cr: 16.8 ± 2.1 μM[ab]<br>Urea: 7.18 ± 2.62 mM[ab]	IS: 183.3 ± 140.5 μM[ab]<br>Cr: 171.1 ±23.8 μM[ab]<br>Urea: 59.9 ± 10.5 mM[ab]<br>84 hours	Both groups n=6	Iwata K. et al., 2007 [95]*
		Serum	IS: 12.5 ± 1.6 μM[a b]<br>Cr: 17.68± 2.92 μM[a]<br>Urea: 7.8 ± 1.18 mM[a]	IS: 156.3 ± 48.8 μM[ab]<br>Cr: 130.83± 43.98 μM[a]<br>Urea: 48.57 ± 11.96 mM[a]<br>72 hours	n=11-17 for Cr/BUN (assuming 11)<br>n=5 per group (for IS)	Morisaki T. et al., 2008 [96]*
		Plasma	Cr: 18.4 ± 1.8 μM[ab]<br>Urea: 9.32 ± 2.13 mM[ab]<br>IS: 7.87 ± 1.12 μM[b]	Cr: 99.9 ± 17.1 μM[ab]<br>Urea: 42.62 ± 4.80 mM[ab]<br>IS: 128.09 ± 23.6 μM[b]<br>72 hours	Both groups n=3-5	Kusumoto M. et al., 2011 [97]*

		Plasma	Cr: 29.17 ± 12.25 μM[a]<br>IS: 4.02 ± 1.63 μM	Cr: 197.1 ± 62.7 μM[a]<br>IS: 151.9 ± 51.3 μM<br>72 hours	Both groups<br>n=3-7	Matsushima A. et al., 2017 [98]*
Wistar	CKD (5/6 nephrectomy)	Serum	HA: 12.3±9.3 μM<br>Cr: 67.01±8.21 μM[a]<br>ClCr: 2.83 ± 0.35 mL/min<br>Urea: 5.5 ± 0.7 mM[a]<br>ClHArenal: 18.1 ± 2.71 mL/min/kg<br>GFR: 2.43 ± 0.697 mL/min/kg	HA: 135±46.5 μM<br>Cr: 167.08±34.2 μM[a]<br>ClCr: 1.58 ± 0.23 mL/min<br>Urea: 15.4 ± 1.8 mM[a]<br>ClHArenal: 2.68 ± 4.6 mL/min/kg<br>GFR: 0.59 ± 0.50 mL/min/kg<br>4 weeks	Normal (n=15-20)<br>CKD (n=15-20)<br>(assuming 15)	Deguchi T. et al., 2005 [99]*
		Plasma	Cr: 24.5 ± 0.01 μM[ab]<br>Urea: 7.05 ± 0.01 mM[ab]<br>IS: 6.88 ± 0.01 μM[b]<br>pCS: 9.88 ± 6.58 μM[b]	Cr: 190.0 ± 75.9 μM[ab]<br>Urea: 45.99 ± 16.86 mM[ab]<br>IS: 183.6 ± 68.5 μM[b]<br>pCS: 129.2 ± 34.57 μM[b]<br>Week 4	Both groups<br>n=12	Sueyoshi M. et al., 2019 [100]*
		Serum	IS: 1.2±0.56 μM<br>Urea: 5.71 ± 5.68 mM[a]<br>Cr: 33.59±32.8 μM[a]<br>ClCr: 2.4 ± 0.48 mL/min	IS: 17.4±2.12 μM<br>Urea: 36.1 ± 13.2 mM[a]<br>Cr: 173.27±35.08 μM[a]<br>ClCr: 0.72 ± 0.56 mL/min<br>10 weeks	Sham (n=7)<br>CRF (n=7)	Shimoishi K. et al., 2007 [101]*
	CKD (3/4 and 5/6 nephrectomy)	Serum	IS: 11.73±6.9 μM[a]<br>Cr: 47.74±8.67 μM[a]<br>ClCr: 1.99 ± 0.78 mL/min	3/4Nx:<br>IS: 43.62±14.8 μM[a]<br>Cr: 68.95±16.76 μM[a]<br>ClCr: 0.88 ± 0.095 mL/min<br>Is not significant<br>6 weeks	Normal (n=24)<br><br>Both CKDs<br>(n=10)	Owada S. et al., 2007 [67]*
				5/6Nx:<br>IS: 77.39±32.6 μM[a]<br>Cr: 137.02±120.2 μM[a]<br>ClCr: 0.45 ± 0.19 mL/min<br>6 weeks		
	Adenine induced	Plasma	IS: 2.35±2.3 μM[a]<br>Cr: 17.1±2.57 μM	IS: 62.85±19.5 μM[a]<br>Cr:83.3±14.5 μM	Control (n=6)<br>Adenine (n=6)	Ali BH. et al., 2019 [68]*

			Urea 3.7 ± 0.49 mM<br>ClCr: 2.2 ± 0.24 mL/min	Urea: 22.0 ± 3.4 mM<br>ClCr: 0.2 ± 0.0 mL/min		
		Plasma	IS: 1.79±1.98[*] μM<br>Cr: 63.09±11.7 μM<br>Urea: 6.36 ± 1.96 mM<br>ClCr: 1.06 ± 0.49 mL/min	IS: 159.55±32.97[*] μM<br>Cr: 205.57±30.5[*] μM<br>Urea: 150.71 ± 7.08 mM<br>ClCr: 0.29 ± 0.098 mL/min<br>29 days	Control (n=6)<br>Adenine (n=6)	Ali BH. et al., 2014 [102]*
		Serum	HA: 6.35±0.96 μM<br>Cr: 32.0±4.5 μM	HA: 40.31±1.98 μM<br>Cr: 140.7±27.5 μM	Both groups (n=6/7)	Huang M. et al., 2018 [103]*
		Plasma	Urea: 22.8 ± 5.1 mM[ab]<br>Cr: 80.6 ± 19.1 μM[ab]<br>ClCr: 0.064 ± 0.018 mL/min[b]<br>IS: 1.67 ± 2.9 μM[ab]	Urea: 77.5 ± 25.5 mM[ab]<br>Cr: 136.8 ± 36.8 μM[ab]<br>ClCr: 0.028 ± 0.009 mL/min[b]<br>IS: 68.2 ± 12.0 μM[ab]<br>5 weeks	Control (n=8)<br>Adenine (n=6)	Suleimani Y. et al., 2015 [104]*
		Plasma	IS: 6.0 ± 4.3 μM[b]<br>pCS: 0.14 ± 0.02 μM[b]<br>HA: 3.32 ± 1.2 μM[b]<br>PS: 3.3±0.5 μM<br>Cr: 21.89 ± 5.05 μM[b]<br>Urea: 6.44 ± 2.14 mM[b]	IS: 190.1 ± 88.98 μM[b]<br>pCS: 27.9 ± 27.5 μM[b]<br>HA: 96.9 ± 70.2 μM[b]<br>PS: 105.2±21.1 μM<br>Cr: 207.9 ± 71.3 μM[b]<br>Urea: 68.2 ± 12.0 mM[b]	Control (n=9)<br>CKD (n=12)	Kucey et al., 2019 [105]*
		Plasma	Urea: 3.0 ± 1.5<br>Cr: 20.5 ± 3.9 μM<br>IS: 4.2 ± 0.73 μM<br>ClCr: 1.77 ± 0.54 mL/min	Urea: 18.0 ± 3.85 mM<br>Cr: 58.2 ± 12.5 μM<br>IS: 34.8 ± 4.12 μM<br>CrCl: 0.44 ± 0.20 mL/min<br>35 days	Both groups n=6	Al Za'abi M. et al., 2021 [106]*
		Urine	Cr: 4258.7 ± 197.2 μM	Cr: 908.3 ± 408.3 μM<br>35 days		
		Plasma	Cr: 17.45 ± 2.55 μM<br>Urea: 3.73 ± 0.61mM<br>ClCr: 2.10 ± 0.29 mL/min<br>IS: 14.4 ± 2.3 μM	Cr: 57.82 ± 9.41 μM<br>Urea: 16.41 ± 2.42 mM<br>ClCr: 0.35 ± 0.049 mL/min<br>IS: 132.6 ± 12.2 μM<br>33 days	Both groups (n=6)	Al Za'abi M. et al., 2021 [107]*

		Urine	Cr: 4808.96 ± 313.98 µM[a]	Cr: 777.92 ± 106.11 µM[a]		
	Cisplatin induced	Plasma	Cr: 20.98 ± 3.92 µM<br>Urea: 6.13 ± 3.77 mM<br>IS: 27.02 ± 5.51 µM[a]<br>ClCr: 2.47 ± 0.69 mL/min	Cr: 141.2 ± 47.08 µM<br>Urea: 42.94 ± 15.4 mM<br>IS: 54.03 ± 6.88 µM[a]<br>ClCr: 0.18 ± 0.07 mL/min<br>4 weeks	Both groups n=6	Al Za'abi M. et al., 2020 [108]*
		Urine	Cr: 5457.2 ± 518.56 µM	Cr: 975.0 ± 123.9 µM		
MICE						
C57BL/6J	CKD (5/6 nephrectomy)	Plasma	IS: 14.54± 5.16 µM[a]<br>Urea: 14.64 ± 1.79 mM[a]	IS: 30.49± 9.85 µM[a]<br>Urea: 25.71 ± 3.9 mM[a]<br>Week 4	Both groups n=10	Ito S. et al., 2013 [109]*
		Plasma	Urea: 12.5 ± 0.7 mM[a]<br>IS: 9.99 ± 1.29 µM[ab]	Urea: 24.3 ± 15.0 mM[a]<br>IS: 49.0 ± 5.36 µM[ab]<br>Week 28-30	Both groups n=12	Nishikawa M. et al., 2015 [110]*
		Serum	Urea: 8.75 ± 0.69 mM[ab]<br>Cr: 31.9 ± 3.6 µM[ab]<br>IS: 31.6 ± 19.9 µM[b]	Urea: 25.02 ± 3.1 mM[ab]<br>Cr: 67.97 ± 25.3 µM[ab]<br>IS: 123.3 ± 85.0 µM[b]<br>Week 6	Both groups n=6	Dou L. et al., 2018 [69]*
		Serum	IS: 8.72 ± 0.67 µM[ab]<br>Cr: 8.84 ± 0.45 µM[ab]	IS: 49.6 ± 9.58 µM[ab]<br>Cr: 27.3 ± 4.7 µM[ab]<br>Week 12	Control n=5<br>CKD n=6	Yamada Y. et al., 2019 [111]*
		Plasma	IS: 2.2 ± 1.1 µM[b]<br>Cr: 58.5 ± 6.1 µM[ab]<br>Urea: 9.1 ± 0.96 mM[ab]	IS: 48.4 ± 16.3 µM[b]<br>Cr: 233.8 ± 92.6 µM[ab]<br>Urea: 80.0 ± 13.0 mM[ab]<br>72 Hours	Control n=4<br>CKD n=8	Yabuuchi N. et al., 2021 [112]*
	Adenine (0.2%) induced renal failure	Plasma	Urea: 10.3 ± 1.2 mM[ab]<br>Cr: 9.0 ± 0.6 µM[b]<br>IS: 16.9 ± 3.0 µM[b]<br>pCS: 0.68 ± 0.001 µM[b]	Urea: 22.5 ± 6.5 mM[ab]<br>Cr: 28.1 ± 4.2 µM[ab]<br>IS: 86.8 ± 17.5 µM[b]<br>pCS: 9.3 ± 6.0 µM[b]	Control n=6<br>CKD n=8	Mishima E. et al., 2018 [113]*
		Urine	IS: 3.2 ± 0.63 µM/mgCr[b]<br>pCS: 0.06 ± 0.01 µM/mgCf[b]	IS: 4.3 ± 0.86 µM/mgCr[b]<br>pCS: 0.74 ± 0.14 µM/mgCr[b]		

		Plasma	Urea: 9.2 ± 1.0 mM[ab]<br>Cr: 11.7 ± 2.4 µM[ab]<br>IS: 4.03 ± 3.5 µM[b]<br>HA: 2.44 ± 3.22 µM[b]	Urea: 29.5 ± 3.1 mM[ab]<br>Cr: 31.2 ± 1.04 µM[ab]<br>IS: 79.3 ± 15.4 µM[b]<br>HA: 25.70 ±5.81 µM[b]	Control n=9<br>CKD n=5	Nanto-Hara F. et al., 2020 [114]*
C57BL/6	CKD (5/6 nephrectomy)	Urine	IS:296 ± 126 µg/day<br>ClCr: 15.3 ± 4.1 µL/min per g	IS: 436 ± 180 µg/day<br>ClCr: 5.6 ± 1.6 µL/min per g<br>Week 25	Both groups n=10	Yamamoto S. et al., 2011 [70]
	CKD (5/6 nephrectomy)	Plasma	IS: 12.79 ± 2.2 µM[ab]<br>Urea: 9.1 ± 1.4 mM[ab]<br>Cr: 56.6 ± 31.9 µM[ab]	IS: 37.1 ± 3.8 µM[ab]<br>Urea: 22.4 ± 7.0 mM[ab]<br>Cr: 86.0 ± 47.4 µM[ab]<br>Week 4	Both groups n=9	Hung S. et al., 2016 [115]*
	CKD (5/6 nephrectomy)	Serum	IS: 7.97 ± 2.5 µM[a]<br>pCS: 3.19 ± 1.4 µM[a]<br>Urea: 9.5 ± 1.3 mM[a]	IS: 15.48± 3.99 µM[a]<br>pCS: 3.72 ± 3.0 µM[a]<br>Urea: 15.9 ± 1.98 mM[a]	Sham n=7<br>CKD n=8	Nakada Y. et al., 2019 [116]*
	CKD (5/6 Nx)	Plasma	Cr: 13.7 ± 0.01 µM[ab]<br>IS: 6.67 ± 2.7 µM[ab]	Cr: 79.6 ± 35.7 µM[ab]<br>IS: 164.3 ± 7.1 µM[ab]<br>20 weeks	Control n=5<br>CKD n=10	Tungasanga S. et al., 2022 [117]*
	Adenine (0.2%) induced renal failure	Serum	Urea: 2.82 ± 0.20 mM[a]<br>Cr: 27.5 ± 3.68 µM<br>IS: 14.5 ± 5.66 µM	Urea: 11.7 ± 2.52 mM[a]<br>Cr: 81.0 ± 12.45 µM<br>IS: 161.8 ± 93.6 µM	Both groups n=8	Makhloufi C. et al., 2020 [118]*
	CKD (5/6 nephrectomy)		IS: 12.9 ± 5.9µM<br>Urea: 3.3 ± 0.3 mM[a]<br>Cr: 32.4 ± 3.1 µM	IS: 75.4 ± 84.3 µM<br>Urea: 8.6 ± 2.5 mM[a]<br>Cr: 55.6 ± 5.9 µM		
	Cisplatin	Plasma	Cr: 3.1 ± 0.6 µM[ab]<br>Urea: 15.5 ± 0.96 mM[ab]<br>IS: 14.67 ± 1.13 µM[ab]	Cr: 47.8 ± 3.8 µM[ab]<br>Urea: 77.3 ± 5.1 mM[ab]<br>IS: 49.3 ± 5.3 µM[ab]<br>4 weeks	Control n=6<br>CKD n=7	Yamakage S. et al., 2021 [119]*
Male ICR	CKD (5/6 Nx)	Plasma	Urea: 7.5 ± 0.4 mMa<br>Cr: 17.68 ± 1.77 µMa<br>IS: 4.7 ± 0.4 µM	Urea: 20.2 ± 3.4 mMa<br>Cr: 70.72 ± 27.40 µMa<br>IS: 39.2 ± 7.7 µM	Not reported<br>ALL DATA ARE ± SEM	Enoki Y. et al., 2017 [120]

B alb/c	CKD (5/6 nephrectomy)	Plasma	pCS:0.76 ± 0.04 µM[ab]<br>Urea: 3.1 ± 0.3 mM[a]<br>Cr: 1.31 ± 0.11 µM	pCS: 2.48 ± 0.17 µM[ab]<br>Urea: 5.9 ± 0.4 mM[a]<br>Cr 9.31 ± 0.33 µM	Both groups n=6	Ni J. et al., 2014 [121]
	CKD (5/6 nephrectomy)	Plasma	pCS: 0.76 ± 0.04 µM[ab]<br>Urea: 3.1 ± 0.3 mM[a]	pCS: 0.72 ± 0.11 µM[ab]<br>Urea: 2.2 ± 0.07 mM[a]		
IQI	CKD UNX (unilateral nephrectomy Adenine (0.2%) induced renal failure	Plasma	IS: 3.68 ± 0.86 µM<br>Cr: 5.06 ± 0.75 µM[ab]<br>Urea: 8.5 ± 1.4 mM[ab]	IS: 20.2 ± 5.8 µM<br>HA: 11.6 ± 5.5 µM<br>Kyn: 0.85 ± 0.03µM<br>Cr: 16.7 ± 3.5 µM[ab]<br>Urea: 19.5 ± 7.0 mM[ab]	Normal n=4<br>CKD n=6	Mishima E. et al., 2017 [39]*
		Urine	IS: 3.24 ± 0.63 µmol/mgCr[b]<br>pCS: 4.7 ± 1.4 µmol/mgCr[b]<br>HA: 6.08 ± 1.2 µmol/mgCr[b]	IS: 4.3 ± 1.0 µmol/mgCr[b]<br>pCS: 4.97 ± 1.4 µmol/mgCr[b]<br>HA: 6.08 ± 7.3 µmol/mgCr[b]		
BALB/c	Adenine (0.175%) induced renal dysfunction	Plasma	Urea: 9.1 ± 1.1 mM[ab]<br>Cr: 17.0 ± 1.9 µM[ab]<br>IS: 1.48 ± 0.01 µM[b]<br>pCS: 19.0 ± 3.8 µM[b]	Urea: 21.3 ± 3.4 mM[ab]<br>Cr: 31.5 ± 5.2 µM[ab]<br>IS: 7.2 ± 5.0 µM[b]<br>pCS: 65.0 ± 13.5 µM[b]	Control n=5<br>CKD n=6	Shiba T. et al., 2018 [122]*
	Adenine (0.2%) induced renal dysfunction			Urea: 47.7 ± 20.3 mM[ab]<br>Cr: 50.5 ± 16.3 µM[ab]<br>IS: 10.68 ± 13.1 µM[b]<br>pCS: 140.87 ± 55.5 µM[b]		
DOG						
Female adult mongrel	Cisplatin	Plasma	HA: 25.67±2.23 µM[a]<br>KA: 5.76±3.84 µM[a]<br>Cr: 83.10±9.72 µM[a]<br>BUN: 16.2 ± 1.3 mg/dL	72 hours post-surgery:<br>HA: 213.212±130.60 µM[a]<br>KA: 137.83±83.01 µM[a]<br>Cr: 963.58±138.79 µM[a]<br>BUN: 156 ± 6.0 mg/dL	n=5	Kawamura M. et al., 1994 [123]

Unknown mix of different strains, both outpatient and in-patient cases	Uremia (by ligation of bilateral renal arteries)<br>Azotaemic<br>AKI<br>CKD of unknown origin	Plasma	IS: 33.77 (IQR: 41.27) µM[a]<br>Cr: 106.1 (IQR: 53) µM<br>BUN: 6.78 (IQR: 6.1) mmol/L<br>(Median+IQR)	Azotaemia:<br>IS: 95.68 (IQR: 138.36) µM[a]<br>Cr: 282.9 (IQR: 415.5) µM<br>BUN: 29.3 (IQR: 47.9) mmol/L	Control n=63<br>Azotaemia n=66<br>CKD n=51<br>AKI n=15	Cheng F. P. et al., 2015 [124]
				AKI:<br>IS: 270.63(IQR: 191.36) µM[a]<br>Cr: 742.6 (IQR: 265.2) µM<br>BUN: 78.7 (IQR: 34.0) mmol/L		
				CKD:<br>IS: 83.02(IQR: 177.72) µM[a]<br>Cr: 185.6 (IQR: 88.4) µM<br>BUN: 37.3 (IQR: 31.0) mmol/L		
Unknown mix of different strains, both outpatient and in-patient cases	CKD of unknown origin<br>CKD of unknown origin	plasma	Median (IQR)	Non-progressive:<br>IS: 3470.76 (3000.74–5487.55) µM[a]<br>Cr: 176.8 (141.4–263) µM	Only uremic : 36 dogs	Chen C. et al., 2018 [46]
				Progressive:<br>IS: 8348.58 (5253.04–16134.33) µM[a]<br>Cr: 203.3 (152.5–267.4) µM		
CAT						
Unknown mix of different strains, both	CKD of unknown origin	plasma		Non-progressive:<br>IS: 45.50 (31.42–59.10) µM[a]	Only uremic : 58 cats	Chen C. et al., 2018 [46]

outpatient and in-patient cases	CKD of unknown origin			Cr: 247.5 (198.9–269.6) µM<br>Progressive:<br>IS: 74.11 (56.75–98.02) µM[a]<br>Cr: 256.4 (212.2–371.3) µM		
Unknown mix of different strains	CKD of unknown origin<br>CKD of unknown origin	serum	IS: 5.636 (0.95-13.41) µM[a]<br>pCS: 15.44 (4.79-38.36) µM[a]<br>Cr: 106.08 (61.88-141.44) µM[a]<br>BUN: 24 (20-38) mg/dl<br><br>Median (range)	CKD stage 2:<br>IS: 15.81 (3.50-48.31) µM[a]<br>pCS: 36.88 (0.18-162.59) µM[a]<br>Cr: 176.80 (141.44-229.84) µM[a]<br>BUN: 43 (20-60) mg/dl<br>CKD stage 3 and 4:<br>IS: 29.95 (4.78-129.45) µM[a]<br>pCS: 28.16 (1.00-187.57) µM[a]<br>Cr: 282.89 (256.36-609.97) µM[a]<br>BUN: 52 (33-98) mg/dl	Healthy n=11<br>CKD 2 n=17<br>CKD 3/4 n=13	Summers S. et al., 2018 [125]
Unknown mix of different strains	CKD of unknown origin<br>CKD of unknown origin<br>CKD of unknown origin<br>CKD of unknown origin	Serum	IS: 7.83 (4.69-9.38) µM[a]<br>Cr: 123.76 (114.92-132.60) µM[a]<br>BUN: 22.5 (19-27) mg/dl<br><br>Median (IQR)	CKD stage 2:<br>IS: 13.65 (9.38 -22.51) µM[a]<br>Cr: 194.48 (176.80-221.00) µM[a]<br>BUN: 28.5 (24-42) mg/dl<br>CKD stage 3:<br>IS: 27.20 (14.54-46.90) µM[a]<br>Cr: 327.09 (291.73-362.45) µM[a]<br>BUN: 52 (38.5-64.5) mg/dl	CKD 2 n=38<br>CKD 3 n=23<br>CKD 4 n=12<br>Healthy n=20	Liao Y. et al., 2019 [126]

				CKD stage 4:<br>IS: 120.54 (98.49-140.71) μM[a]<br>Cr: 570.19 (459.69-698.37) μM[a]<br>BUN: 106.5 (77.8-130.3) mg/dl		
Unknown mix of different strains	CKD of unknown origin	Plasma	IS: 69.41 (57.69) μM<br>BUN: 8.2 (4.3) mM<br>Cr: 132.6 (17.7) μM<br>Median (IQR)	IS: 98.49 (88.64) μM<br>BUN: 19.3 (33.0) mM<br>Cr: 229.9 (194.5) μM	CKD n=69<br>Control n=16	Cheng F.P. 2015 [124]
Goat						
Female Dutch White adult goats (capra aegagrus hircus)	CKD of unknown origin	Plasma	IS: 1.39±0.50 μM<br>IAA: 0.46±0.10 μM<br>Kyn: 3.15±1.04 μM<br>KA: 0.01±0.01μM<br>PCG: 0.52±0.14 μM<br>pCS: 37.00±18.00 μM<br>HA: 43.00±20.00 μM<br>Cr: 65.00±5.60 μM	IS: 69.00±76.00 μM<br>IAA: 0.67±0.35 μM<br>Kyn: 1.67±1.18 μM<br>KA: 0.09±0.07μM<br>PCG: 47.00±64.00 μM<br>pCS: 985.00±843.00 μM<br>HA: 817.00±913.00 μM<br>Cr: 841.00±584.00 μM	n=5 pre<br>n=11 post	Van Gelder et al., 2021 [127]
Pig						
female Yorkshire pigs	Subtotal renal artery embolization and gentamicin (acute-on-chronic)	Plasma	IS: 0.55±0.33 μM<br>IAA: 0.74 ±1.21 μM<br>Kyn: 0.75±0.30 μM<br>KA: 0.01±0.00μM<br>PCG: 4.49±3.52 μM<br>pCS: 0.24±0.26 μM<br>HA: 10.09±4.15 μM<br>Cr: 103.00±14.00 μM	IS: 26.09±27.72 μM<br>IAA: 0.33±0.18 μM<br>Kyn: 1.01±0.45 μM<br>KA: 0.01±0.00μM<br>PCG: 43.03±77.01 μM<br>pCS: 4.85±5.26 μM<br>HA: 63.76±98.99 μM<br>Cr: 932.00±470.00 μM	n=5	De Vries et al., 2022 [128]

[a] = Unit changed

[b] = Estimated from graphical presentation

*= Included in the analyses

AKI= Acute Kidney Injury, BUN= Blood Urea Nitrogen, CKD= Chronic Kidney Disease, Cr= Creatinine, HA= Hippuric Acid, IAA=indole 3-Acetic Acid, IQR= Inter Quartile Range, IS= Indoxyl Sulfate, KA= Kynurenic Acid, Kyn= Kynurenine, pCS= P-Cresyl Sulfate, PS= Phenyl Sulfate
